# MIA-Jet: Multi-scale Identification Algorithm of Chromatin Jets

**DOI:** 10.1101/2025.08.27.672730

**Published:** 2025-09-01

**Authors:** Sion Kim, Minji Kim

**Affiliations:** 1 Gilbert S. Omenn Department of Computational Medicine and Bioinformatics, University of Michigan, Ann Arbor, MI, 48109, USA.; 2 Department of Electrical and Computer Engineering, University of Michigan, Ann Arbor, MI, 48109, USA.

**Keywords:** 3D genome mapping, chromatin jets, ridge detection, scale-space, chromatin loop formation, DNA replication

## Abstract

The mammalian genome is organized into large-scale chromosome territories, compartments, domains, and at the smallest scale, chromatin loops and stripes. The newest element is a chromatin jet, a diffused line perpendicular to the main diagonal in the Hi-C contact map, which was reported in quiescent mammalian lymphocytes supporting a two-sided symmetric cohesin loop extrusion model. A similar structure is observed in Repli-HiC data, where relatively thin and straight chromatin fountains indicate coupling of DNA replication forks. However, the precise biological implications of these jet-like structures are unknown due to the limitations in computational methods. We developed MIA-Jet, a multi-scale ridge detection algorithm that can accurately detect jets of variable lengths, widths, and angles. When tested on Hi-C, Repli-HiC, ChIA-PET, ChIA-Drop, and Micro-C data in mouse, human, roundworm, and zebrafish cells, MIA-Jet outperformed existing methods. In human cells, jets were enriched in cohesin loading sites and early replication initiation zones. Applying MIA-Jet to Hi-C data generated from protein-degraded cells revealed that jets are dependent on cohesin but not YY1, and jet signals are strengthened after depleting WAPL. We envision MIA-Jet to be broadly applicable to any 3D genome mapping data, thereby providing new insights into the functional roles of chromatin jets.

## INTRODUCTION

The advancement in high-throughput three-dimensional (3D) genome mapping technologies revealed that the mammalian genome is highly organized within the 3D nuclear space. At the largest scale, chromosomes occupy distinct territories, which encompass compartments and topologically associating domains (Lieberman-Aiden et al., 2009; Dixon et al., 2012). At a smaller scale, various protein factors mediate chromatin loops to bridge distal genomic loci together (Fullwood et al., 2009; Li et al., 2012; Tang et al., 2015; Weintraub et al., 2017; Mumbach et al., 2016; Grubert et al., 2020) where loops are sometimes accompanied by architectural stripes (Vian et al., 2018). Each of these chromatin structures has potential roles in cellular processes. In particular, chromatin loops are considered the basic unit of higher order genome organization and are extensively studied (Phillips-Cremins et al., 2013; Braccioli and de Wit, 2019; Davidson et al., 2023). For instance, more than 22 computational tools have helped to annotate and characterize chromatin loops in 3C data (Chowhury et al., 2024). It is now known that cohesin extrudes DNA until it is blocked by a zinc finger protein CTCF, which binds to specific DNA motifs. Most of the chromatin loops are anchored at a pair of convergent CTCF binding motifs (Rao et al., 2014; Tang et al., 2015), suggesting that the orientation of binding motifs is related to the biophysical mechanism of chromatin loop formation. Notably, perturbing CTCF sites alters genome organization and has implications in cancer and genetic disorders (Ushiki et al., 2021; Kubo et al., 2021), highlighting the importance of chromatin loops in maintaining the normal function of cells.

Understanding the fundamental biophysical principle of chromatin loop formation is critical for cataloging their functional roles in cellular processes such as gene transcription. However, the extrusion mechanism remains unsettled. Cohesin was proposed to extrude DNA loops symmetrically in a two-sided manner *in vitro* through biochemical and single-molecule imaging methods (Davidson et al., 2019; Kim et al., 2019), yet a recent *in vitro* study supported an asymmetric one-sided extrusion model instead (Barth et al., 2025). The problem is equally perplexing *in vivo*. Architectural stripes (Vian et al., 2018) imply that cohesin extrusion occurs asymmetrically at one side of the CTCF loop anchoring sites, while chromatin jets reported in Hi-C data of quiescent mouse cells (Guo et al., 2022) and *in silico* simulations (Banigan et al., 2020) posit that cohesin extrudes in a two-sided symmetric fashion upon being loaded onto the DNA by loading factors including NIPBL. These isolated results have raised debates regarding the model of cohesin extrusion *in vivo* (Drayton et al., 2022). A key question is whether chromatin jets are widely observed in mammalian cells or are specific to quiescent mouse cells, but it is unclear as the 37 jets were manually annotated without a computational algorithm to automatically detect jets.

Subsequent studies also revealed jet-like patterns—sometimes referred to as fountains or plumes—in multiple species and 3D genome mapping technologies. A preprint described that in *C. elegans*, chromatin fountains at active enhancer sites that disappear upon cohesin cleavage (Luthi et al., 2023). Another study using auxin-inducible degron system confirmed that jets are dependent on cohesin and that they emerge from NIPBL binding (cohesin loading) sites; depleting a cohesin unloading factor WAPL resulted in the extension of jets (Kim and Ercan, 2025), akin to the extended loops observed in human cells (Haarhuis et al., 2017). Performing Hi-C experiments in zebrafish embryos showed that fountains emerge selectively at enhancers upon zygotic genome activation and the authors of this preprint attribute the findings to two-sided, desynchronized loop extrusion by cohesin upon loading onto the DNA (Galitsyna et al., 2023). Jets have also been reported in fungi (Shao et al., 2024) and mouse embryonic stem cells (Liu et al., 2025). The aforementioned results are derived from Hi-C data analysis and unanimously suggest that jets are: 1) found in various species, 2) often associated with active enhancer or cohesin loading sites, and 3) dependent on cohesin. Recently, a new replication-associated in situ Hi-C method (Repli-HiC) was developed to enrich for chromatin interactions involving nascent DNA (Liu et al., 2024), similar to ChIA-PET (Fullwood et al., 2009), HiChIP (Mumbach et al., 2016) or PLAC-seq (Fang et al., 2016) that enrich for protein-mediated interactions. By applying Repli-HiC to human cells, the authors identified two classes of chromatin fountains: Type-I fountains (median length of 160 kb) favoured at early replication initiation zones, and Type-II fountains (median length of 1 Mb) associated with replication termination (Liu et al., 2024). Collectively, these results provide convincing evidence that the chromatin jets observed by Guo et al. in 2022 may be a genome-wide phenomena across various technologies and species.

However, current progress is impeded by a lack of computational tools to identify and quantify jets in an unbiased manner genome wide. The 37 jets in mouse cells were manually annotated by hand (Guo et al., 2022), while Repli-HiC fountains were identified by a local enrichment method called Fun (Liu et al., 2024). Other studies have computationally identified jets by kernel-based similarity methods Fontanka (Galitsyna et al., 2023), Chromosight (Matthey-Doret et al., 2020), and Luthi et al., 2023. These methods require well-defined kernels, which may require hand annotation by users. The method used by Luthi et al., 2023 also mandates cohesin-depleted Hi-C data as an input to calculate the expected background distribution. Most importantly, current methods are limited in identifying jets of fixed width and angle, thereby posing a computational challenge in finding diffused or curved jets reported in Guo et al., 2022. Kernel-based methods also assume fixed length, which is not suitable for Repli-HiC fountains as Type-I and Type-II fountains differ in length (median of 160 kb vs. 1 Mb). Therefore, existing computational methods cannot be broadly applied to identify jets of various forms. As a result, it is challenging to answer important biological questions, such as: if jets are found in all cells, species, across technologies; whether jets coincide with cohesin loading sites for loop extrusion and/or DNA replication origins; if depleting protein factors result in changes in the number, strength, and length of jets.

To both overcome the limitations of existing algorithms and to answer biological questions, we developed MIA-Jet, a multi-scale ridge detection algorithm to accurately identify jets of variable length, width, and angle. It is inspired by the earlier success of computer vision-based methods MUSTACHE (Roayaei Ardakany et al., 2020), SIP (Rowley et al., 2020), and Stripenn (Yoon et al., 2022) to call chromatin loops or architectural stripes. When benchmarked on Hi-C data in quiescent mouse cells and Repli-HiC data in human K562 cells, MIA-Jet outperformed existing algorithms Fun and Fontanka with respect to known features such as NIPBL binding intensity and replication signal, as well as the jet signals. MIA-Jet identified jets across various technologies—in situ Hi-C, intact Hi-C, Repli-HiC, ChIA-PET, ChIA-Drop, Micro-C—in human, mouse, *C. elegans*, and zebrafish (Supplementary Table S1; Reiff et al., 2022; Luo et al., 2020; Rao et al., 2017; Rao et al., 2014; Kim et al., 2024; Liu et al., 2024; Kieffer-Kwon et al., 2017; Guo et al., 2022; Hsieh et al., 2022; Kim et al., 2025; Morao et al., 2022; Wike et al., 2021). Compared to the random background locations, jets identified by MIA-Jet had high insulation scores, were enriched in NIPBL binding signal and enhancer marks, and were at or near early DNA replication initiation zones. Finally, using publicly available depletion Hi-C and Micro-C datasets, we show that depleting cohesin eliminates jets, while degrading WAPL results in stronger and longer jets; CTCF and YY1 seem to have minimal impact on jet formation and maintenance. We envision MIA-Jet to be instrumental in answering key biological questions in the 3D genomics field as it is versatile and applicable to accommodate a wide range of genome mapping technologies, species, and types of jets.

## RESULTS

### Overview of the MIA-Jet algorithm

**Figure 1:**
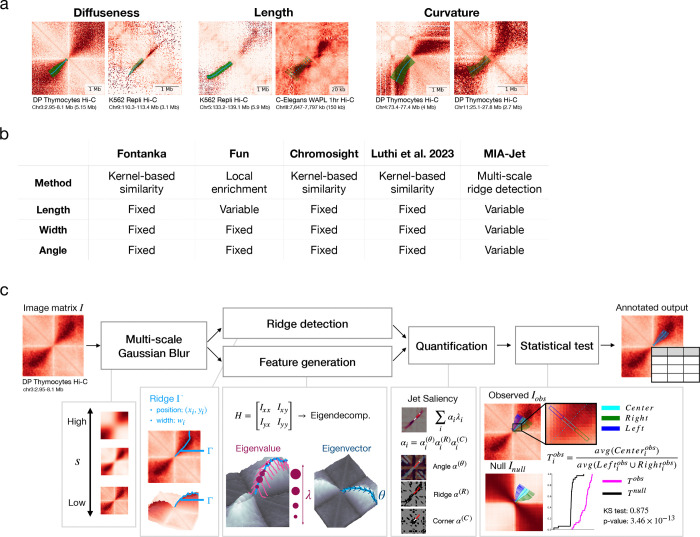
Overview of MIA-Jet algorithm. **(a)** 2D contact maps of observed/expected at 25 kb resolution for all regions except *C. Elegans* at 2 kb resolution with MIA-Jet output annotation in the bottom left triangle. Cyan lines: ridge coordinates, green rectangles: ridge widths. **(b)** Summary table of available jet calling algorithms Fontanka, Fun, Chromosight, Luthi et al., 2023, and MIA-Sig. **(c)** MIA-Jet pipeline detailing the major components of the algorithm. The input .hic file is converted to an image I, which undergoes Gaussian blurring at multiple scales s∈S. For feature generation, the image Hessian is computed per pixel to obtain ridge strength (eigenvalue) and angle (eigenvector). Features are combined with ridges from ‘ridge detection’ to rank each ridge by *jet saliency*, a weighted sum of the ridge strength where weights can softly impose constraints, such as pointing in a desired angle range. Finally, a statistical test is performed on a local enrichment ratio compared to that of the null model. The output is a table with jet position, width and quantification metrics. See also Figure S1.

Chromatin jets pose unique computational challenges that are distinct from other chromatin features such as TADs, loops, and stripes, for which there already exist a plethora of available callers (Xu et al., 2024; Chowdhury et al., 2024; Raffo and Paulsen 2023). First, jets can be diffused as observed in DP thymocyte Hi-C data or sharp in the case of K562 Repli-HiC data (Figure 1a, ‘Diffuseness’), resulting in variable widths. Second, jets vary in length: some jets are as large as 4 Mb while others are small in the order of 50 kb (Figure 1a, ‘Length’). Third, unlike architectural stripes that are horizontal or vertical, jets may extrude at variable angles and exhibit curvature as the jet protrudes (Figure 1a, ‘Curvature’). Existing computational tools cannot accommodate all three features of jets. Fun (Liu et al., 2024) scans through the genome and computes local enrichment of jet-like structures to identify Type-I and Type-II fountains in Repli-HiC data by allowing users to specify the expected length of the fountains but yields jets of fixed width and angle (Figure 1b). (Galitsyna et al., 2023), Chromosight (Matthey-Doret et al., 2020), and Luthi et al., 2023 requires users to provide the mask, which is either averaged hand-annotated regions or a binary mask, to compute the similarity between the kernel and sliding window across the main diagonal. As these methods rely heavily on the kernel, they can only identify specific types of jets and potentially miss others.

Here, we present MIA-Jet, a multi-scale ridge detection algorithm that can accurately identify jets of variable lengths, widths, and angles. We leverage concepts from image processing to define chromatin jets as *ridges*, which are curves on the image surface, oriented to maximize the second derivative (Haralick, 1983). Utilizing local second order derivatives, we bypass the need to create kernels. Ridge detection is combined with scale-space for multi-scale ridge detection (Lindeberg, 1998). MIA-Jet first applies Gaussian blur at multiple scales to the input Hi-C matrix to find jets of various sizes (Figure 1c). The algorithm then detects ridge positions by the *CurveTracing* ImageJ plugin. Separately, MIA-Jet computes the image Hessian H and generates the following features: ridge strength λ, ridge direction θ, ridge condition R, corner condition C and smallest eigenvalue ψ (Figure S1). Ridges then undergo trimming and are quantified based on their scale space features, enabling metrics to filter out false positives and rank ridges based on specific properties (Figure S1, ‘Quantification’). Finally, the algorithm tests for significance of the observed ridge compared to a background null model and outputs annotated jets with genomic coordinates, width to measure the diffuseness of jets (Figure 1a, lower triangle of 2D contact maps), and a tunable measure of jet strength, *jet saliency*. The MIA-Jet algorithm is implemented into a publicly available python program. Algorithmic details are provided in the Methods section.

### MIA-Jet outperforms existing methods on quiescent mouse cells Hi-C data

**Figure 2:**
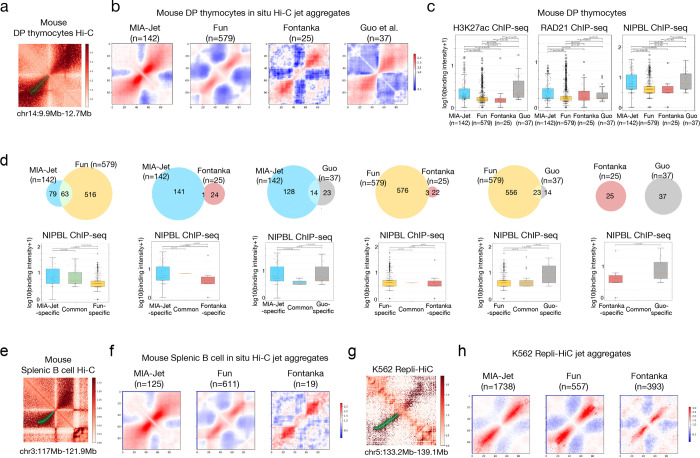
Benchmark of MIA-Jet, Fun, Fontanka on mouse Hi-C data and human Repli-HiC data. **(a)** 2D contact map of mouse DP thymocyte Hi-C data with MIA-Jet position annotation (cyan lines) and width (green rectangles). **(b)** Aggregate contacts at jet locations identified by MIA-Jet, Fun, Fontanka, and Guo et al., 2022, in mouse DP thymocytes in situ Hi-C data. The two-sided Wilcoxon rank-sum p-values comparing columns 1 and 2, 1 and 3, 1 and 4, 2 and 3, 2 and 4, 3 and 4: H3K27ac 1.5e-19 (***), 4.7e-7 (***), 0.0067 (**), 0.069, 1.8e-12 (***), 1.5e-7 (***); RAD21 1.1e-8 (***), 0.23, 0.38, 0.42, 0.0063 (**), 0.62; NIPBL 1.6e-12 (***), 0.00056 (***), 0.81, 0.58, 2e-5 (***), 0.0018 (**) **(c)** Boxplots of log10(binding intensity+1) of H3K27ac, RAD21, and NIPBL ChIP-seq in mouse DP thymocytes at jet locations identified by each method, where n is number of called jets. **(d)** Comparison of jets identified by pairs of methods. Venn Diagram shows the number of jets common between the two methods in the middle, and those not shared (‘-specific’) on the left and right. The log10(binding intensiy+1) of NIPBL ChIP-seq signal is shown for each of the three categories in a boxplot. The two-sided Wilcoxon rank-sum p-values comparing columns 1 and 2, 1 and 3, and 2 and 3, respectively: MIA-Jet vs Fun 0.026 (*), 1e-12 (***), 5.9e-5 (***); MIA-Jet vs Fontanka 0.73, 0.00081 (***), 0.13; MIA-Jet vs Guo et al. 5.6e-5 (***), 0.58, 0.0034 (**); Fun vs Fontanka 0.95, 0.54, 0.8; Fun vs Guo et al. 0.79, 0.0038 (**), 0.04 (*); Fontanka vs Guo et al. 0.0018 (**) **(e)** 2D contact map of mouse splenic B cell Hi-C data with MIA-Jet annotation in cyan lines and green rectangles. **(f)** Aggregation of contacts over jet locations identified by each method in mouse splenic B cell Hi-C data, with n denoting the number of jet regions. **(g)** An example of K562 Repli-HiC jet (or a fountain) identified by MIA-Jet. **(h)** K562 Repli-HiC contacts aggregated at jet regions identified by MIA-Jet, Fun, and Fontanka. See also Figure S2.

To evaluate the performance of MIA-Jet, we benchmarked existing methods Fun and Fontanka in DP thymocytes Hi-C data. Fontanka was selected among the kernel-based methods as the authors had previously compared their results with Chromosight. A representative jet called by the MIA-Jet program is shown (Figure 2a). The top 5 jets ranked by jet saliency in MIA-Jet include known Guo et al. jets as ranks 1 and 3, with ranks 2, 4, and 5 also exhibiting jet-like structures (Figure S2a, top panel). Top 5 jets ranked by ‘SoN’ (signal over noise) in Fun reported a mixture of jets, stripes and dots (Figure S2a, middle panel) whereas Fontanka mostly reported compartments or domains (Figure S2a, bottom panel). A major advantage of MIA-Jet is its ability to capture the width or diffuseness of jets. For example, a jet found on chromosome 14 is annotated with narrow widths from the origin and gradually increasing off diagonal (Figure 2a). Genome-wide aggregation of jets identified by each method showed that MIA-Jet had the strongest jet-like signals over 142 jets while Fun identified 579 jets of predefined widths and lengths; Fontanka and Guo et al. only had 25 and 37 jets (Figure 2b). Of the three methods, MIA-Jet had highest binding signals in H3K27ac, RAD21, and NIPBL, confirming the features of jets reported in Guo et al. (Figure 2c). As NIPBL binding is indicative of cohesin loading sites from which jets were shown to originate in quiescent mouse cells, we used it as a proxy for a likelihood of a true jet. While Fun and Fontanka identified 516 and 24 jets missed by MIA-Jet, respectively, NIPBL binding at Fun-specific and Fontanka-specific jet origins were lower than that of MIA-Jet-specific jets (Figure 2d). When compared against 37 jets by Guo et al., MIA-Jet had comparable NIPBL binding intensity to that of Guo et al., and higher Jaccard index (0.085) than Fun (0.038) and Fontanka (0) (Figure 2d).

Mouse splenic B cell Hi-C data also had a similar jet-like structure at a location showcased by Guo et al. in DP thymocytes, but the extent to which jets are conserved between the two cell types was unknown due to the lack of jet annotations. By applying MIA-Jet to both cell types, we identified 32 common jets and 110 DP thymocyte-specific, and 93 splenic B cell-specific jets with each categories all exhibiting strong jet-like structures (Figure S2b). When Hi-C contacts were aggregated at jet regions identified in DP thymocytes, the splenic B cell data showed weaker yet visible jet signals (Figure S2c). An example of a slightly curved jet is shown for splenic B cell Hi-C data, further highlighting MIA-Jet’s unique capability and generalizability (Figure 2e). Fun and Fontanka also identified 611 and 19 jets in splenic B cell, yet their aggregated jet signal was much weaker than that of MIA-Jet (Figure 2f).

These results confirm that MIA-Jet can identify diffused, angled jets present in quiescent mouse cells and recapitulate epigenomic features therein.

### MIA-Jet can identify fountains in K562 Repli-HiC data

The Type-I and Type-II fountains reported in Liu et al., 2024 were generally narrow and had a consistent perpendicular extrusion from the main diagonal. To evaluate MIA-Jet’s performance in identifying this different type of jets, we ran MIA-Jet, Fun, and Fontanka on K562 Repli-HiC data. A representative Type-II jet called by MIA-Jet is shown (Figure 2g). Top 5 jets from MIA-Jet mostly had fountain-like structures, while Fun and Fontanka included false positives in the top rankings (Figure S2d). In contrast to the variable widths reported in mouse quiescent cells (Figure 2a, Figure 2e), MIA-Jet accurately quantified and captured the narrow and consistent width in K562 Repli-HiC data (Figure 2g). When we aggregated Hi-C contacts over the jets called by each method, MIA-Jet and Fun had comparably strong signals, but MIA-Jet identified 3-fold more jets than Fun (1738 vs. 557) (Figure 2h). Of note, MIA-Jet identified 1345 distinct jets that are elongated, while Fun-specific jets resemble dots rather than jets and are relatively weaker in strength (Figure S2e, top panel). Both MIA-Jet and Fun outperformed Fontanka in showing strong jet-like signals (Figure S2e, middle, bottom panels). MIA-Jet reported jets of variable lengths ranging from 100 kb to 4 Mb with a median of 750 kb, while Fun was mostly limited to jets of length 500 kb to 1.5 Mb with a median of 991 kb, likely because each run requires a fixed length (Figure S2f). In terms of run time, MIA-Jet, Fun, and Fontanka each took 8, 6, 26 minutes on DP thymocyte Hi-C data and 45, 9, 26 minutes on K562 Repli-HiC data, respectively (Figure S2g). Peak memory consumption was highest for Fontanka at 47.4 GB and less than 10 GB for MIA-Jet and Fun (Figure S2h).

These benchmarking results suggest that MIA-Jet is accurate and computationally efficient, motivating us to utilize MIA-Jet to comprehensively characterize jets across various technologies and cell types to uncover biological features of jets.

### Repli-HiC has longer and stronger jets than Hi-C

The Repli-HiC method enriches for chromatin interactions associated with nascent DNA among all interactions present in Hi-C experiments, resulting in a subset of contacts. Thus, in theory, Hi-C experiments should also have jets, which are simply buried among all other chromatin structures, as shown in exemplary locations in Liu et al., 2024. We sought to systematically compare the presence, strengths, and overlaps of jets in Repli-HiC, in situ Hi-C, and intact Hi-C in K562 cells by calling jets via MIA-Jet in an unbiased manner.

**Figure 3:**
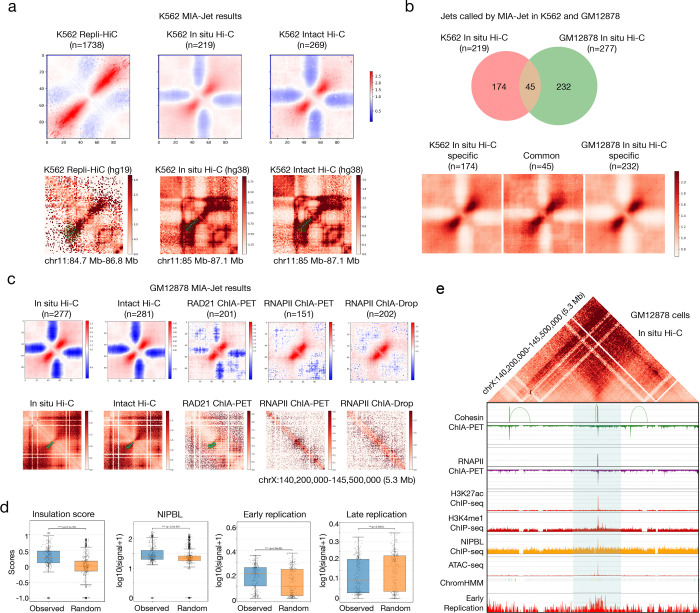
MIA-Jet results in K562 and GM12878 human cell lines. **(a)** Top: Aggregation of 2D contacts of jets identified by MIA-Jet in K562 Repli-HiC, in situ Hi-C, and intact Hi-C, where n is the number of jets in each dataset. Bottom: Exemplary location of jets identified in all three datasets with MIA-Jet results annotated in cyan and green. **(b)** A Venn diagram of jets comparing K562 in situ Hi-C and GM12878 in situ Hi-C with aggregation of contacts for each of the three categories (K562-specific, Common, GM12878-specific) below. **(c)** Top: Aggregation of 2D contacts of jets identified by MIA-Jet in GM12878 cells for 5 datasets: in situ Hi-C, intact Hi-C, RAD21 ChIA-PET, RNAPII (RNA Polymerase II) ChIA-PET, and RNAPII ChIA-Drop. Bottom: A 5.3 Mb region on chromosome X with MIA-Jet annotations in green, if detected. **(d)** Boxplots of insulation scores 2.4e-30 (***) and log10(signal intensity+1) of NIPBL ChIP-seq 2.5e-9 (***), Early replication from Repli-seq 7.8e-8 (***), and Late replication 0.0061 (**) from Repli-seq. Observed: 277 jets identified by MIA-Jet in the GM12878 in situ Hi-C data. Random: 277 random regions (see **Methods**). **(e)** The same 5.3 Mb jet region as presented in panel **(c)**, with GM12878 in situ Hi-C contacts on the top, loops and binding peaks of cohesin and RNAPII ChIA-PET below it. The read coverage of H3K27ac, H3K4me1, NIPBL ChIP-seq, and ATAC-seq are displayed below, with chromHMM state denoting enhancer (yellow) at the jet origin. Bottom track shows early replication signal in Repli-seq data. The jet region identified by MIA-Jet is highlighted in light blue. See also Figure S3.

As expected, Repli-HiC had 6 to 8-fold more jets (n=1738) than in situ Hi-C (n=219) and intact Hi-C (n=269), and had the strongest jet signals (Figure 3a, top panel). Repli-HiC jets were also longer (median = 750 kb; mean = 1 Mb) than in situ Hi-C (median = 500 kb; mean = 547 kb) and intact Hi-C (median = 500 kb; mean = 612 kb) (Figure S3a). A jet found in Repli-HiC data is also recapitulated in the in situ Hi-C and intact Hi-C data, with the latter two also exhibiting stripes and TADs (Figure 3a, bottom panel). To answer whether such conservation of jets across technologies is common, we computed the overlap between each set of jets found in a pair of data. Most of in situ Hi-C (92 of 217) and intact Hi-C (116 of 266) jets were also identified in Repli-HiC data, yielding a precision of 0.42 and 0.44, respectively, for in situ Hi-C and intact Hi-C (Figure S3b). There were 100 jets that are shared between in situ Hi-C and intact Hi-C (Jaccard index of 0.26), suggesting that Hi-C experiments—whether in situ or intact—largely capture jets at similar location.

By contrast, the jets identified in GM12878 cells generally did not overlap those in K562 cells using the same in situ Hi-C technique, with only 45 common jets between them and a Jaccard index of 0.1 (Figure 3b, top panel). However, all three categories of K562-specific, common, and GM12878-specific jets had similar strengths in signal (Figure 3b, bottom panel). As the GM12878 cell line has a rich set of publicly available genomic data, we next sought to identify genomic features of jets identified across various 3D genome mapping technologies in GM12878.

### Jets are enriched at cohesin loading sites and are associated with early replication

We first asked whether enriching for chromatin interactions mediated by protein factors retains jets present in Hi-C experiments. In particular, we chose a cohesin subunit RAD21 as jets may arise from cohesin extrusion, and RNA Polymerase II (RNAPII) as jets were shown to be enriched at active enhancers. While RAD21 ChIA-PET, RNAPII ChIA-PET, RNAPII ChIA-Drop data exhibit jet-like signals (Figure 3c, top panel), their jets are much smaller (median of 300–350 kb) than Hi-C jets (median of 500 kb) (Figure S3c). At a strong jet location on chromosome X, MIA-Jet identified diffused jets in both in situ Hi-C, intact Hi-C, and RAD21 ChIA-PET, but did not report any jets in RNAPII ChIA-PET and RNAPII ChIA-Drop data, implying that RNAPII is not involved in forming this particular jet (Figure 3c, bottom panel). Computing overlap of jets between all pairs of datasets revealed interesting patterns (Figure S3d): in situ and intact Hi-C have the highest overlap (Jaccard index of 0.325), and both Hi-C data have substantial overlap with RAD21 (Jaccard indices 0.216, 0.199) but not with RNAPII (0.163, 0.151); however, RAD21 also has high overlap with RNAPII (0.262, 0.229). This result implies that RAD21 may capture two types of jets: those observed in Hi-C and those observed in RNAPII. Our previous result showing that cohesin has multifaceted roles in forming structural loops with CTCF and transcriptional loops with RNAPII (Kim et al., 2024) corroborates with the findings reported here.

We next used jets identified by MIA-Jet (‘Observed’) to characterize their genomic features compared to the random regions (‘Random’) (see Methods). Across all platforms, jets had higher insulation and compartment scores than random regions (Figure 3d, S3e, S3f), potentially suggesting that jets reside in active regions within TADs. As shown in mouse DP thymocytes, human GM12878 cells also had jets with enriched NIPBL binding signals (Figure 3d, S3g) which may coincide with cohesin loading sites related to extrusion activities. Jets identified in Hi-C, ChIA-PET, and ChIA-Drop data were also enriched in early replication but not in late replication signals (Figure 3d, S3h), which is consistent with the observations in Repli-HiC data on K562 cell-line (Liu et al., 2024). When we examined the jet shown in Figure 3c, the jet origin shows binding of cohesin (with small loops) and RNAPII in ChIA-PET data. This region is also enriched in H3K27ac, H3K4me1, NIPBL, and ATAC-seq signals as well as being annotated as active enhancer by chromHMM (Figure 3e). In addition, Repli-seq data indicates that the jet origin is related to early DNA replication signals.

Based on these observations, we next focused on the 277 jets identified in the GM12878 in situ Hi-C data as it does not have any bias towards protein-enriched or replication-enriched activities (e.g., ChIA-PET or Repli-HiC). While these epigenomic features hint that jet origins are likely cohesin loading sites, as was assumed by Guo et al. in concluding that jets arise due to cohesin extrusion, we adopted a more rigorous definition of true loading sites. In addition to having high 1-dimensional binding of active marks and NIPBL, we impose that cohesin loading sites are not bound by CTCF, far from nearest CTCF binding sites, and is encapsulated by at least one strong convergent CTCF loop (see Methods). Doing so revealed that jets are more likely to originate from cohesin loading or anchoring sites than random regions, but explains only 32 of 277 jets (Figure S3i). Likewise, jets are more likely to be encapsulated by a strong convergent CTCF loop than by chance (Figure S3j). Finally, chromatin states at jet origins were mostly enhancers or promoters (175 of 277 jets), while random regions only had 103 enhancer/promoter marks and were mostly heterochromatin (Figure S3k).

### Jets are abolished without cohesin and elongated after WAPL depletion

The auxin-inducible degron (AID) system (Natsume et al., 2016) is a powerful tool for rapidly depleting protein factors for studying their function *in vivo*. Applying these tools to human and mouse cells revealed that depleting cohesin results in loss of all chromatin loops (Rao et al., 2017) while degrading WAPL created extended longer loops (Haarhuis et al., 2017). However, little is known about the effect of protein degradation on chromatin jets, mainly due to the lack of computational tools to identify and quantify jets genome-wide. Here, we apply MIA-Jet to the publicly available Hi-C or Micro-C data generated in human, mouse, and *C. elegans* cells with depletion of cohesin, CTCF, YY1, or WAPL.

**Figure 4:**
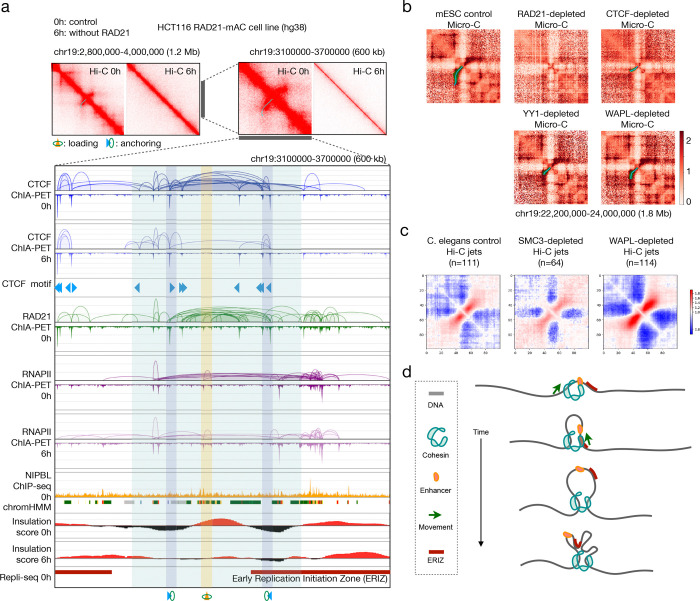
Effects of protein depletion on chromatin jets. **(a)** Data shown for HCT116 RAD21-mAC cell line engineered to rapidly deplete RAD21 with auxin treatment. ‘0h’: without auxin treatment (before depletion; control), ‘6h’: 6 hours of auxin treatment (RAD21 depletion). A 600 kb region encompasses a jet called by MIA-Jet, highlighted in light blue. CTCF anchoring sites (with convergent motifs) are highlighted in dark blue, while cohesin loading site is in yellow. CTCF, RAD21, RNAPII (RNA Polymerase II) ChIA-PET data have loops and binding coverage tracks. NIPBL ChIP-seq is shown below, with chromHMM states denoting enhancers (yellow), promoters (yellow) and transcription (green). Insulation scores from Hi-C data and Repli-seq DNA replication initiation zones are shown below. **(b)** An example of curved jets in mouse embryonic stem cells (mESC) Micro-C data in protein (RAD21, CTCF, YY1, WAPL) depleted cells, with MIA-Jet annotation and contact map at 25 kb resolution. **(c)** Aggregation of 2D contacts at jets identified by MIA-Jet in C. elegans control, SMC3-depleted, and WAPL-depleted cells, where n denotes the number of jet regions. **(d)** A proposed model of chromatin jet formation, where cohesin first loads onto the DNA at active enhancer sites near early replication initiation zone (ERIZ), then alternately extrudes from the left and right strands until the extruded strands fold among themselves. See also Figure S4.

One of the most widely known perturbation studies in the 3D genomics field is the RAD21 depletion in HCT116 colorectal cancer cell line. While previous studies focused on chromatin loops (Rao et al., 2017), we applied MIA-Jet to identify jets in the same dataset. In a region on chromosome 19, a jet is identified in Hi-C data before depleting RAD21 (‘0h’) but the jet structure disappears in the Hi-C data generated after 6 hours of auxin treatment for RAD21 depletion (‘6h’) (Figure 4a). When we zoom into the jet region highlighted in light blue, we note that jet origin (highlighted in yellow) was encapsulated by a strong convergent CTCF/RAD21 loop (with its anchors in dark blue), which is attenuated upon removal of RAD21 (‘CTCF ChIA-PET 6h’). The jet origin also coincided with small loops and bindings by RAD21 and RNAPII as well as NIPBL binding and enhancer sites. Notably, the insulation score was high in ‘0h’—as was observed in GM12878 in situ Hi-C jets—but much weaker without RAD21 (‘6h’). Finally, the jet origin was near the early replication initiation zone (ERIZ) defined by Repli-seq data. Similar patterns are found in another exemplary jet (Figure S4a). These results confirm that the high insulation score, NIPBL binding, enhancer/promoter states, and early replication initiation zone characterized genome-wide in GM12878 in situ Hi-C jets are not only recapitulated in HCT116 in situ Hi-C jets but also may be responsible for the loss of jets upon depleting RAD21.

Micro-C was developed to generate higher resolution contacts than Hi-C, thereby improving the signals of smaller-scale structures such as chromatin jets. In the control mouse embryonic stem cells (mESC) Micro-C data, a clear curved jet is identified by MIA-Jet (Figure 4b). However, this structure is demolished or attenuated upon RAD21 or CTCF depletion, resulting in no jet or a small jet found by MIA-Jet. By contrast, depleting YY1 had minimal effect on the jet, while WAPL depletion resulted in a visibly longer jet (Figure 4b). This pattern is confirmed genome-wide for *C. elegans* Hi-C data, where MIA-Jet identified 111 jets in the control, a two-fold less 64 jets in SMC3-depleted cells, and the strongest 114 jets in WAPL-depleted cells (Figure 4c). Of note, jets are longer in WAPL-depleted cells (median = 65 kb; mean = 69.2 kb) than the control (median = 50 kb; mean = 57.2 kb). Aggregating contacts at the 111 jet locations found in control confirmed that the jet signals disappear after depleting SMC3, and the jets are stronger and longer without WAPL (Figure S4b). A specific example supports this genome-wide analysis (Figure S4c). Finally, MIA-Jet identified 95 jets in zebrafish embryo Hi-C data (Figure S4d) of median length 800 kb, with exemplary straight and curved jets displayed (Figure S4e).

## DISCUSSION

We described MIA-Jet and demonstrated its utility in accurately identifying jets of various diffuseness, lengths, widths, and angles. Notably, it does not require training steps or kernels, thereby allowing users to detect jets in a wide range of settings in 3D genome mapping data. MIA-Jet is also one of the few publicly available software for calling jets. Recently, an improved version of the Fun algorithm, called Fun2 (Zhangding et al., 2025), appeared on a preprint server, and the Hi-C data in mouse embryonic stem cells of WAPL, CTCF, and cohesin also became publicly available (Liu et al., 2025). Comparing MIA-Jet to Fun2 and applying MIA-Jet to the mouse ESC Hi-C data would be an interesting future direction. In addition, as Fun2 can identify both jets and stripes, it would also be fun to test whether MIA-Jet can detect stripes by modifying the angle range parameter and imposing stricter conditions on the widths.

There are limitations of MIA-Jet in its current form. First, MIA-Jet is dependent on several parameters that have been finetuned for each genomic technologies and organisms, such as the resolution and window size. Second, while MIA-Jet uses the scale-space framework, it does not perform explicit scale tracking of key structures, such as ridges (jets or stripes) and corners (Hi-C checkerboard pattern and loops), which would help to reduce false positives while enabling the ability to capture significant variation in scale within a single chromatin jet.

The MIA-Jet findings offer answers to several biological questions, yet also pose new ones. Our motivation for developing MIA-Jet was to determine whether jets arise as part of the two-sided cohesin loop extrusion model or as DNA replication. However, to our surprise (and slight disappointment), the results supported both scenarios: jets were enriched at cohesin loading sites (Figure 3d, S3i) as well as early replication initiation zones (ERIZ) (Figure 3d, 4a, S4a). One interpretation is that cohesin extrusion and DNA replication processes are interdependent on each other, but such hypothesis requires additional experimental validations. We posit that cohesin loads onto the DNA near the ERIZ and extrudes in a two-sided asymmetric manner—sometimes reeling the right strand or the left strand—and the extruded DNA strands crumble up to form a TAD-like structure (Figure 4d). Intriguingly, it is unclear which environmental forces or protein factors stops jet extrusion, analogous to the role of convergent CTCF on stopping chromatin loops. In addition, jets seem to gradually fade and increase in diffuseness as they move away from the main diagonal. Both the underlying molecular mechanism and biophysical principles for such patterns are yet to be explored. By leveraging publicly available Hi-C data, one can also ask if certain cell types encompass more jets, and whether jets are conserved across cell lineages or are cell-type-specific. Integrative analyses of all chromatin structures, ranging from large-scale TADs and compartments to finer-scale loops, stripes, and jets would be worthwhile. As the 3D genomics field ventures into this new area of chromatin biology, we envision MIA-Jet to provide the important first steps towards understanding biological implications of chromatin jets and beyond.

## METHODS

### Notation

Let the input Hi-C contact matrix be $A\in R^{p\times p}$. Let h be the binned window size parameter. Then, the extracted rectangular image is $I\in R^{m\times n}$ where $m\approx \frac{1}{\sqrt{2}}h$ and $n\approx p\sqrt{2}$ (Image generation). Let $S=\left\{s_{1},s_{2},\ldots s_{k}\right\}$ denote the set of standard deviations of Gaussian blurring. Let $I^{\left(s\right)}=g_{s}\;*\;I$, s∈S denote the blurred image, where $g_{s}$ is the 2D Gaussian kernel with standard deviation s and * is the convolution operator. From here on, we refer to the standard deviation s∈S as *scale*. Let $K_{x^{a}y^{b}}$ denote the discrete ath order x-derivative and bth order y-derivative kernel. Define the *scale space derivative kernel* (Lindeberg, 1998)

$${𝒟}_{x^{a}y^{b}}^{\gamma ,s}=s^{\gamma (a+b)}K_{x^{a}y^{b}},$$

which normalizes the derivative kernel $K_{x^{a}y^{b}}$. Here, γ∈[0,1] is the user-defined *gamma* parameter. Given an image $I^{\left(s\right)}$ blurred at scale s, let the generated *feature maps* at scale s refer to the following: ridge strength map $\Lambda^{\left(s\right)}\in R^{m\times n}$, the ridge angle map $\Theta^{\left(s\right)}\in R^{m\times n}$, the ridge condition map $R^{\left(s\right)}\in R^{m\times n}$ and corner condition map $C^{\left(s\right)}\in R^{m\times n}$ and the smallest eigenvalue map $\Psi^{\left(s\right)}\in R^{m\times n}$. Let a single ridge be defined as $Z_{j}$. The following definitions refer to a single jth ridge $Z_{j}$ detected at an image blurred at scale $s^{*}\in S$.

- Let $N_{j}$ be the number of points in the ridge.
- Let the position of each point of the ridge be defined as $\left(p_{1},p_{2},\ldots ,p_{N_{j}}\right)$, where point $p_{i}=\left(y_{i},x_{i}\right)$ is a coordinate containing the row index $y_{i}$ and column index $x_{i}$ with respect to the rectangular image I. Assume that the ridge points are not significantly curved and are ordered according to genomic distance from the main diagonal, which ensuring the inequality $x_{1}\geq x_{2}\geq \cdots \geq x_{N_{j}}$. Then, ridge point index i=1 is closest to the main diagonal and $i\in \left\{1,2,\ldots ,N_{j}\right\}$ increases with genomic distance from the main diagonal (see Figure S1 ‘1^st^ position’).
- Let the widths of the ridge be defined as $\left(w_{1},w_{2},\ldots ,w_{N_{j}}\right)$ where $w_{i}$ is the width of point $p_{i}$ in pixels.
- Denote the ridge strength $\left(\lambda_{1}^{\left(s^{*}\right)},\lambda_{2}^{\left(s^{*}\right)},\ldots ,\lambda_{N_{j}}^{\left(s^{*}\right)}\right)$, where $\lambda_{i}=\Lambda^{\left(s^{*}\right)}\left[y_{i},x_{i}\right]$ is the ridge strength at the ridge coordinates $p_{i}=\left(y_{i},x_{i}\right)$. The superscript ($s^{*}$) is dropped unless ambiguous, referring to the image scale of the detected ridge. Similarly, define the ridge angles $\left(\theta_{1}^{\left(s^{*}\right)},\theta_{2}^{\left(s^{*}\right)},\ldots ,\theta_{N_{j}}^{\left(s^{*}\right)}\right)$, where $\theta_{i}=\Theta^{\left(s^{*}\right)}\left[y_{i},x_{i}\right]$, ridge conditions $\left(r_{1},r_{2},\ldots ,r_{N_{j}}\right)$, $r_{i}=R^{\left(s^{*}\right)}\left[y_{i},x_{i}\right]$, corner conditions $\left(c_{1}^{\left(s^{*}\right)},c_{2}^{\left(s^{*}\right)},\ldots ,c_{N_{j}}^{\left(s^{*}\right)}\right)$, $c_{i}=C^{\left(s^{*}\right)}\left[y_{i},x_{i}\right]$, and smallest eigenvalues $\left(\Psi_{1}^{\left(s^{*}\right)},\Psi_{2}^{\left(s^{*}\right)},\ldots ,\Psi_{N_{j}}^{\left(s^{*}\right)}\right)$, $\Psi_{i}=\Psi^{\left(s^{*}\right)}\left[y_{i},x_{i}\right]$.

### Inputs

The MIA-Jet program takes input a .hic file. At a minimum, the path to the .hic file, the chromosome, Hi-C resolution, and experiment type (“hic” or “replihic”) must be specified. Bash scripts are provided to call MIA-Jet as separate processes across all chromosomes. Detailed parameter specification is in Supplementary Table S1 ‘MIA-Jet Parameters’ (see also https://github.com/minjikimlab/miajet/tree/main ).

### Image generation

#### Image for ridge characterization I

The input Hi-C contact map $A\in R^{p\times p}$ is read in using the python package *hicstraw* using the specified data type, normalization, and resolution. The columns and rows which sum to 0 are removed. If the data type is “observed”, then a log-transform is applied to reduce dynamic range. The contact map is then clipped such that intensity values less than the *im_vmin*-th percentile are set to the *im_vmin*-th percentile and values greater than the *im_vmax*-th percentile are set to the *im_vmax*-th percentile. If the *im_vmin*-th percentile and the *im_vmax*-th percentile are identical, then we perform no clipping. The contact map is min-max normalized to be between 0 and 1 using *cv.normalize*. Finally, the contact map is rotated by 45° using *scipy.ndimage.rotate* with *mode=rotation_padding* user parameter. The upper-triangle region along the main diagonal is extracted to yield a rectangular matrix $I\in R^{m\times n}$, where $m\approx \frac{1}{\sqrt{2}}h$ and $n\approx p\sqrt{2}$.

#### Images for p-value computation $I_{\mathrm{obs}},\;I_{\mathrm{null}}$

Two additional Hi-C images are generated to compute p-values: the observed and null images (see Figure 1 ‘Statistical Test’). Both $I_{\mathrm{obs}},\;I_{\mathrm{null}}$ are derived from the same contact map where the data type of the Hi-C is fixed to be “observed”, which we denote as $A_{p-val}$. This contact map undergoes log-transform followed by percentile clipping and 0–1 normalization as described above. $I_{\mathrm{obs}}$ is simply the rectangle extracted from the rotated $A_{p-\mathrm{val}}$, whereas $I_{\mathrm{null}}$ is the rectangle extracted from the Hi-C correlation matrix, computed by $\frac{1}{p}{\left(A_{p-val^{\prime }}\right)}^{T}A_{p-val}^{\prime }$, where ${A_{p-val}}^{\prime }$ is obtained by z-standardizing the columns of $A_{p-val}$.

#### Image for corner condition $\tilde{I}$

An additional Hi-C image is generated to compute the corner condition map. We first read in the contact map by fixing the data type parameter to be “oe”, which we denote as $\tilde{A}$. The columns and rows which sum to 0 for A are removed. Then, the off-diagonal region beyond the specified window size is set to 0. This contact map undergoes log-transform followed by percentile clipping and 0–1 normalization as described above. $\tilde{I}$ is given by the rectangle extracted from Hi-C correlation matrix of $\tilde{A}$.

### Gaussian blurring

#### Generating scales considered for Gaussian blurring

Let the *jet_widths* user parameter be $b_{lb}$, $b_{ub}$, which is the lower and upper jet widths that the program can detect (in pixels). If the *jet_widths* parameter is specified, then we convert $b_{lb}$, $b_{ub}$ from widths in pixels to scales using the following equation (https://imagej.net/plugins/ridge-detection):

$$s=\frac{b}{2\sqrt{3}}+0.5,\;b\in \left\{b_{lb},b_{ub}\right\}$$

obtaining $s_{lb}$, $s_{ub}$. We then determine the number of scales n_scales by uniformly sampling log-space as follows:

$$n_{\mathrm{\_scales}}=min\left\{max\left\{round\left(\frac{\ell_{1}\;-\;\ell_{0}}{\delta }\right),3\right\},30\right\}$$

where $\ell_{0}={log}_{1.5}\left(s_{lb}\right)$ and $\ell_{1}={log}_{1.5}\left(s_{ub}\right)$ and scale resolution δ. We fix the log base as 1.5 and scale resolution δ=0.25. The set of scales S is then generated by a log-space grid using *numpy.logspace* with $start=\ell_{0}$, $stop=\ell_{1}$, num=n_scales, *base=1.5*. Optionally, the *scale_range* parameter can be specified which receives the set S from the user directly as a list of numbers. If neither *jet_widths* nor *scale_range* is specified, then S is defined by a default log-space grid with *start=1*, *stop=7* and *num=24*. Once S is defined, we ensure that no scale is too large for the image dimensions by filtering out scales which exceed $s_{max}=\frac{min\{m,n\}}{4}$.

### Ridge characterization

#### Ridge detection

For the ridge detection module, MIA-Jet calls the ImageJ *CurveTrace* plugin (https://github.com/ekatrukha/CurveTrace ) for each s∈S using the following parameters: Line width=*s*, Split lines at junction=0, Correct line position, and Compute line width=2.5, *Upper=thresholds[1]*, and *Lower=thresholds[0]*, where *thresholds* is the user-defined parameter of the MIA-Jet program, specifying the lower and upper thresholds of the hysteresis thresholding scheme for ridge detection. If *thresholds* is None, then the threshold is estimated for each scale s∈S using the formula provided in the ImageJ Ridge Detection plugin (https://imagej.net/plugins/ridge-detection ). The lower and upper contrast values are estimated by the 25^th^ and 90^th^ percentile of the non-zero values of the image. For each scale, we save the Hi-C image with the ridges overlaid and a results table containing the detected ridge coordinates, angle and width. The result table is combined across scales.

#### Ridge Processing

The result table undergoes additional processing. Angles are converted to degrees and wrapped from [0,360°] to [0, 180°]. If a ridge is not significantly curved, it is sorted according to genomic distance from the main diagonal by ensuring $x_{1}\geq x_{2}\geq \cdots \geq x_{N_{j}}$, where $x_{i}$ is the column index of each ridge point. Ridge points which lie outside the Hi-C contact map or are in *rem_k_strata* pixel within the main diagonal are removed. After this step, ridges that only has one point remaining are removed, i.e., ensuring $N_{j}>1$. Optionally, we also remove entire ridges if it is too distant from the main diagonal. Specifically, any ridge must have at least some part of it within *root_within* pixels to the main diagonal, or equivalently that $\underset{i\in \left\{1,2,\ldots N_{j}\right\}}{max}\;x_{i}\leq root\_within$ is satisfied. Otherwise, the entire ridge is discarded.

In the image generation step for I, MIA-Jet removed the columns and rows which summed to 0. To ensure that the ridge coordinates align with true genomic locations, the ridge coordinates are appropriately shifted. Let the shifted coordinates for the jth ridge $Z_{j}$ be denoted as $\left(p_{1}^{\prime },p_{2}^{\prime },\ldots ,p_{N_{j}}^{\prime }\right)$, where point $p_{i}^{\prime }=\left(y_{i}^{\prime },x_{i}^{\prime }\right)$ is the shifted coordinate. Both the shifted and unshifted coordinates are stored.

#### Feature generation

For each s∈S, the image I is convolved with the Gaussian kernel to obtain $I^{\left(s\right)}$. The blurred image $I^{\left(s\right)}$ is convolved with scale space derivative kernels ${𝒟}_{x^{a}y^{b}}^{\left(\gamma ,s\right)}$, which are normalized derivative kernels,

$$\begin{array}{c} I_{x}^{\left(s\right)}={𝒟}_{x}^{\left(\gamma ,s\right)}\;*\;I^{\left(s\right)},\;I_{y}^{\left(s\right)}={𝒟}_{y}^{\left(\gamma ,s\right)}\;*\;I^{\left(s\right)},\\ I_{xx}^{\left(s\right)}={𝒟}_{xx}^{\left(\gamma ,s\right)}\;*\;I^{\left(s\right)},\;I_{yy}^{\left(s\right)}={𝒟}_{yy}^{\left(\gamma ,s\right)}\;*\;I^{\left(s\right)},\;I_{xy}^{\left(s\right)}={𝒟}_{xy}^{\left(\gamma ,s\right)}\;*\;I^{\left(s\right)}. \end{array}$$

If γ is set appropriately (e.g. γ∈[0.5,1]), the normalization ensures that the true scale of ridges can be identified as the local maxima of the ridge strength map over scale. This property is important for the quantification metrics (see Quantification). From here, the largest eigenvalue of the image Hessian provides the ridge strength map, and the corresponding eigenvector yields the normal direction of the ridge angle map. For i∈{1,2,…,m}, j∈{1,2,…n}, define the image Hessian

$$H_{i,j}^{\left(s\right)}=\left(\begin{array}{ll} I_{xx}^{\left(s\right)}[i,j] & I_{xy}^{\left(s\right)}[i,j]\\ I_{xy}^{\left(s\right)}[i,j] & I_{yy}^{\left(s\right)}[i,j] \end{array}\right),$$

the *ridge strength map*

$$\Lambda^{\left(s\right)}[i,j]=max\left\{\underset{v}{max}\;\frac{v^{T}H_{i,j}^{\left(s\right)}v}{v^{T}v},0\right\}$$

the *ridge angle map*

$$\Theta^{\left(s\right)}[i,j]=\left(arctan2\left(v_{y}^{*},v_{x}^{*}\right)+90^{{}^{\circ}}\right)mod{\;180}^{{}^{\circ}},\;\mathrm{where}\;v^{*}=arg\;\underset{v}{max}\;\frac{v^{T}H_{i,j}^{\left(s\right)}v}{v^{T}v},$$

and the *smallest eigenvalue map*

$$\Psi^{\left(s\right)}[i,j]=\underset{v}{min}\;\frac{v^{T}H_{i,j}^{\left(s\right)}v}{v^{T}v}.$$

Boolean conditions for ridge condition maps and corner condition maps additionally require the gradient magnitude, which is defined as follows,

$$I_{v}^{\left(s\right)}[i,j]=\sqrt{{\left(I_{x}^{\left(s\right)}[i,j]\right)}^{2}+{\left(I_{y}^{\left(s\right)}[i,j]\right)}^{2}}$$

and scale space directional derivatives in the first principal curvature direction $I_{p}^{\left(s\right)}$, $I_{pp}^{\left(s\right)}$ and the second principal curvature direction $I_{q}^{\left(s\right)}$, $I_{qq}^{\left(s\right)}$ (see analytical expressions in Lindeberg, 1998). Then, for i∈{1,2,…,m}, j∈{1,2,…n} the *ridge condition map* (Haralick, Watson, and Laffey, 1983) is given by

$$R^{\left(s\right)}[i,j]=\left\{\begin{array}{llll} 1, & I_{v}^{\left(s\right)}[i,j]>0, & I_{p}^{\left(s\right)}[i,j]=0, & I_{pp}^{\left(s\right)}[i,j]<0\\ 1, & I_{v}^{\left(s\right)}[i,j]>0, & I_{q}^{\left(s\right)}[i,j]=0, & I_{qq}^{\left(s\right)}[i,j]<0\\ 1, & I_{v}^{\left(s\right)}[i,j]=0, & I_{pp}^{\left(s\right)}[i,j]<0, & I_{qq}^{\left(s\right)}[i,j]=0\\ 1, & I_{v}^{\left(s\right)}[i,j]=0, & I_{qq}^{\left(s\right)}[i,j]<0, & I_{pp}^{\left(s\right)}[i,j]=0\\ 0, & \mathrm{otherwise} & & \end{array}.\right.$$

For the corner condition map, the same procedure is applied to obtain the image derivatives and image gradient using the corner image $\tilde{I}$ as opposed to I. For notational simplicity, we refer to the corner image gradient as ${\tilde{I}}_{v}^{\left(s\right)}$ and directional derivatives ${\tilde{I}}_{pp}^{\left(s\right)}$, ${\tilde{I}}_{qq}^{\left(s\right)}$. Then, for i∈{1,2,…,m}, j∈{1,2,…n} the *ridge corner map* (saddle point in Haralick, Watson, and Laffey, 1983) is given by

$$C^{\left(s\right)}[i,j]=\left\{\begin{array}{ll} 1, & {\tilde{I}}_{v}^{\left(s\right)}[i,j]=0,\;{\tilde{I}}_{pp}^{\left(s\right)}[i,j]\cdot {\tilde{I}}_{qq}^{\left(s\right)}[i,j]<0\\ 0, & \;\mathrm{otherwise}\; \end{array}.\right.$$

To compute the derivatives and Gaussian blur, we rely on the discscsp.computeNjetfcn function from the *pyscsp* python package (https://github.com/tonylindeberg/pyscsp), which was modified to enable different convolution padding modes. The *convolution_padding* user parameter controls the padding mode.

### Trimming

The default *thresholds* parameter supplied to the ImageJ ridge detection is not dependent on image statistics. As a result, ridges may extend into noisy regions or through spurious structures, such as compartments. This problem especially prominent in Hi-C data, where other structures such as loops, TADs or compartments may elicit a response as ridges, particularly at high scales. To address this issue, the MIA-Jet program trims ridges based on three different criteria.

Let the ridge being trimmed be $Z_{j}$ detected at scale $s^{*}\in S$. Assume that the ridge positions $i\in \left\{1,2,\ldots ,N_{j}\right\}$ are ordered according to genomic distance from the main diagonal. Recall the ridge strength $\left(\lambda_{1},\lambda_{2},\ldots \lambda_{N_{j}}\right)$, ridge angles $\left(\theta_{1},\theta_{2},\ldots ,\theta_{N_{j}}\right)$, ridge conditions $\left(r_{1},r_{2},\ldots ,r_{N_{j}}\right)$, corner conditions $\left(c_{1},c_{2},\ldots c_{N_{j}}\right)$, and smallest eigenvalues $\left(\psi_{1},\psi_{2},\ldots ,\psi_{N_{j}}\right)$ at the ridge coordinates.

#### Corner trimming

Define the discard set $B_{C}\left(Z_{j}\right)$ as positions of the ridge where a corner is detected

$$B_{C}\left(Z_{j}\right)=\left\{i\in \left\{1,2,\ldots ,N_{j}\right\}|c_{i}^{\left(s\right)}=1,\;\forall s\in S\right\}.$$

The corner condition is given by the saddle point definition in (Haralick, Watson, and Laffey, 1983), which resulted in many potential corners. Thus, a corner must not only be detected at the detected scale of the ridge $s^{*}\in S$ but across all scales. The index $i_{C}^{*}$ at which corner trimming occurs is the position closest to the main diagonal

$$i_{C}^{*}=minB_{C}\left(Z_{j}\right).$$

We keep the ridge positions $\left\{1,2,\ldots ,i_{C}^{*}-1\right\}$ and remove $\left\{i_{C}^{*},i_{C}^{*}+1,\ldots ,N_{j}\right\}$.

#### Angle trimming

Let the user parameter *angle_range* be [$\theta_{lb}$, $\theta_{ub}$]. Define the discard set $B_{\theta }\left(Z_{j}\right)$ as positions of the ridge where the angle does not lie in $\left[\theta_{lb},\theta_{ub}\right]$

$$B_{\theta }\left(Z_{j}\right)=\left\{i\in \left\{1,2,\ldots ,N_{j}\right\}|\theta_{i}^{\left(s^{*}\right)}\notin \left[\theta_{lb},\theta_{ub}\right]\right\}.$$

The index $i_{\theta }^{*}$ at which angle trimming occurs is the position closest to the main diagonal

$$i_{\theta }^{*}=min\;B_{\theta }\left(Z_{j}\right).$$

We keep the ridge positions $\left\{1,2,\ldots ,i_{\theta }^{*}-1\right\}$ and remove $\left\{i_{\theta }^{*},i_{\theta }^{*}+1,\ldots ,N_{j}\right\}$.

#### Smallest eigenvalue trimming

Define the set of local maxima of smallest eigenvalues

$$ℳ=\left\{i\in \left\{2,\ldots N_{j}-1\right\}|\psi_{i}>\psi_{i-1},\psi_{i}>\psi_{i+1}\right\}.$$

Let index $i_{\mathrm{peak}}$ be the first non-negative local maximum if there is a local maximum. Specifically,

$$i_{\mathrm{peak}}=\left\{\begin{array}{rr} min\left\{i\in ℳ|\psi_{i}\geq 0\right\}, & \left\{i\in ℳ|\psi_{i}\geq 0\right\}\neq \emptyset \\ arg\underset{i\in \left\{1,\ldots ,N_{j}\right\}}{max}\;\psi_{i}, & ℳ=\emptyset \\ 1, & \;\mathrm{otherwise}\;\mathrm{(i.e.}\;\mathrm{all}\;\mathrm{local}\;\mathrm{maxima}\;\mathrm{are}\;\mathrm{negative)}\; \end{array}.\right.$$

If there are no local maxima, then the global maximum is used, and if all peaks are negative then the $i_{\mathrm{peak}}$ is set to the first position. Intuitively, $i_{\mathrm{peak}}$ is intended to represent one coherent ridge. If there is a switch in the sign after this point, it is indicative that the ridge has extended into other structures. The index $i_{\psi }^{*}$ at which trimming occurs is the first index at or after $i_{\mathrm{peak}}$ where the smallest eigenvalue turns negative,

$$i_{\psi }^{*}=min\left\{i\in \left\{i_{\mathrm{peak}},i_{\mathrm{peak}}+1,\ldots ,N_{j}\right\}|\psi_{i}<0\right\}.$$

We keep the ridge positions $\left\{1,2,\ldots ,i_{\psi }^{*}-1\right\}$ and remove $\left\{i_{\psi }^{*},i_{\psi }^{*}+1,\ldots ,N_{j}\right\}$.

Each trimming criteria can be toggled independently based on the *corner_trim*, *angle_trim*, and *eig2_trim* parameters respectively. If the trim parameter is greater than 1, then it is interpreted as the minimum ridge length in bins (i.e. any ridge cannot be trimmed such that its length is smaller than the parameter value). If the parameter is in [0,1], then it is interpreted as the minimum fraction of the ridge length. For instance, *angle_trim* = 0.5 means that angle trimming cannot occur in the first half of the ridge. After trimming, we remove ridges which only has one point.

#### Whitespace trimming

In image generation, columns and rows of I which sum to 0 are removed. Ridge detection coordinates on I then undergo shifting such that ridge coordinates align with genomic positions. As a result, some ridges appear to “jump” between zero regions (e.g. extending through unmapped regions). This is reasonable if the unmappable region is small, but if it extends several megabases, then it is unlikely to reflect a true continuation of a chromatin jet. To trim these ridges, we first define the vector of differences between unshifted coordinates $x_{i}$ and shifted coordinates $x_{i}^{\prime }$ for ridge $Z_{j}$

$$d\left(Z_{j}\right)=\left[x_{1}-x_{1}^{\prime },x_{2}-x_{2}^{\prime },\ldots ,x_{N_{j}}-x_{N_{j}}^{\prime }\right].$$

We now define relative shifts that are invariant to any pre-existing global shifts

$$\overset{‾}{d}\left(Z_{j}\right)=\left[0,\left|\left(x_{2}-x_{2}^{\prime }\right)-\left(x_{1}-x_{1}^{\prime }\right)\right|,\left|\left(x_{3}-x_{3}^{\prime }\right)-\left(x_{1}-x_{1}^{\prime }\right)\right|,\ldots ,\left|\left(x_{N_{j}}-x_{N_{j}}^{\prime }\right)-\left(x_{1}-x_{1}^{\prime }\right)\right|\right].$$

Define the discard set $B_{0}\left(Z_{j}\right)$ as positions of the ridge where there is a relative shift of 5 bins or greater

$$B_{0}\left(Z_{j}\right)=\left\{i\in \left\{1,2,\ldots ,N_{j}\right\}|\bar{d_{i}}\left(Z_{j}\right)>5\right\}.$$

The index $i_{0}^{*}$ at which whitespace trimming occurs is the position closest to the main diagonal

$$i_{0}^{*}=min\;B_{0}\left(Z_{j}\right).$$

For whitespace trimming, it is important to trim the side of the ridge where the whitespace occurs. To detect this, let the estimated background intensity of the image $v^{\left(25th\right)}$ be the 25^th^ percentile of the positive pixel values in I. If the image intensity value at ridge positions $\left\{1,\ldots ,i_{0}^{*}-1\right\}$ are all less than $v^{\left(25th\right)}$ or $\left|\left\{1,\ldots ,i_{0}^{*}-1\right\}\right|=1$, then we trim $\left\{1,\ldots ,i_{0}^{*}-1\right\}$ and keep $\left\{i_{0}^{*},\ldots ,N_{j}\right\}$. Conversely, if the image intensity value at ridge positions $\left\{i_{0}^{*},\ldots ,N_{j}\right\}$ is all less than $v^{\left(25\mathrm{th}\right)}$ or $\left|\left\{i_{0}^{*},\ldots ,N_{j}\right\}\right|=1$, then we trim $\left\{i_{0}^{*},\ldots ,N_{j}\right\}$ and keep $\left\{1,\ldots ,i_{0}^{*}-1\right\}$. After trimming, we remove ridges which only has one point, i.e., ensuring $N_{j}>1$.

### Quantification

Ridges detection results often have a high number of false positives. The quantification step aims to assign metrics to each ridge $Z_{j}$ to remove false positives in downstream filtering steps. In addition, the *jet saliency* metric is assigned to each ridge to generate a final ranking, allowing users to have flexibility in emphasizing specific attributes of the ridge, such as the desired orientation.

#### Normalized Entropy

The normalized entropy measure aims to quantify the presence of conflicting structures in scale space. It is possible that there are two ridges in one location of the Hi-C image. For example, consider a stripe on the border of a TAD. At a small scale, the stripe may register as a ridge, whereas at a larger scale the TAD may present itself as a large ridge (Supplementary Figure S1). To filter out such false positives, we require methods to quantify when there are multiple structures existing at different scales.

Let the current ridge be $Z_{j}$. We compute the mean ridge strength across the scales. For each ridge position $i\in \left\{1,2,\ldots N_{j}\right\}$, define

$${\overset{‾}{\lambda }}_{i}=\frac{1}{\left|S\right|}\sum_{s\in S}\lambda_{i}^{\left(s\right)},\;\mathrm{where}\;\lambda_{i}^{\left(s\right)}=\Lambda^{\left(s\right)}\left[y_{i},x_{i}\right].$$

If there is a unique structure in scale space, we expect the mean ridge strength to be similar across the ridge positions. To quantify this, we bin the mean ridge strength values to form a histogram. We utilize *numpy.histogram* with *range=*(*points_min*, *points_max*) and *bins=num_bins*, where *points_min*, *points_max* and *num_bins* are MIA-Jet parameters. Alternatively, it is possible to specify the bin size instead of the number of bins via the *bin_size* parameter. Let the number of bins be q. The histogram is then normalized to sum to 1, representing a probability mass function (PMF) of mean ridge strength values, denoted by $P=[P(1),P(2),\ldots ,P(q)]\in \Delta_{q}$, where $\Delta_{q}$ is the q-simplex. We compute the normalized Shannon Entropy $H_{\mathrm{norm}}(P):\Delta_{q}\to [0,1]$ to quantify the variation of mean ridge strength values

$$H_{\mathrm{norm}}(P)=-\frac{1}{{log}_{2}(q)}\sum_{k=1}^{q}P(k){log}_{2}(P(k)).$$

Normalized entropy $H_{\mathrm{norm}}(P)$ close to 0 suggests a delta-like PMF P, which occurs if the mean ridge strength values are similar. Conversely, a value near 1 captures the case where there is high variation in mean ridge strength, suggesting the existence of multiple structures.

#### Normalized RMSE

The normalized RMSE quantifies abrupt changes in scale. Guo et al. observed 37 jets in mouse DP thymocytes. These jets exhibit diffuseness as they extrude from the main diagonal. In scale space, this corresponds to a slow increase in scale along the ridge position $\left\{1,2,\ldots ,N_{j}\right\}$ ordered in genomic distance from the main diagonal. Ridges that exhibit rapid variation in scale is suggestive of false positives, where a single ridge may go across multiple differentially sized structures, such as TADs, stripes and loops. To quantify this, we compute the expected scale at each position of the ridge. We first define a PMF of ridge strength Q(i) for each position $i\in \left\{1,2,\ldots ,N_{j}\right\}$,

$$Q_{i}=\left[Q_{i}\left(s_{1}\right),Q_{i}\left(s_{2}\right),\ldots ,Q_{i}\left(s_{k}\right)\right]\;\mathrm{where}\;Q_{i}\left(s\right)=\frac{1}{\sum_{u\in S}\;\lambda_{i}^{\left(u\right)}}\lambda_{i}^{\left(s\right)},s\in S.$$

The $PMF\;Q_{i}(s)$ represents the probability that there is a ridge at scale s∈S in position $i\in \left\{1,2,\ldots ,N_{j}\right\}$. Then, the expected scale is computed for each position $i\in \left\{1,2,\ldots ,N_{j}\right\}$

$${\overset{‾}{s}}_{i}=\sum_{s\in S}sQ_{i}(s).$$

A cubic polynomial $\left({\overset{ˆ}{s}}_{1},{\overset{ˆ}{s}}_{2},\ldots ,{\overset{ˆ}{s}}_{N_{j}}\right)$ is fit on the expected scales $\left({\overset{‾}{s}}_{1},{\overset{‾}{s}}_{2},\ldots ,{\overset{‾}{s}}_{N_{j}}\right)$ using *numpy.polyfit*. The normalized root mean square error (RMSE) is given by

$$RMSE_{\mathrm{norm}}=\frac{1}{N_{j}}\sqrt{\sum_{i=1}^{N_{j}}\;\frac{{\left({\overset{‾}{s}}_{i}\;-\;{\overset{ˆ}{s}}_{i}\right)}^{2}}{N_{j}}}$$

A reasonable assumption is that the Guo et al. chromatin jets exhibit a slow increase in scale which can be well represented by a cubic polynomial (Supplementary Figure S1).

#### Jet saliency

Jet saliency is a weighted aggregate of the ridge strength $\left(\lambda_{1},\lambda_{2},\ldots ,\lambda_{N_{j}}\right)$, where the weights enable flexibility in emphasizing particular characteristics of the ridge $Z_{j}$. Let the detected scale of ridge be $s^{*}\in S$. Let the user parameter *angle_range* be $\left[\theta_{lb},\theta_{ub}\right]$. We then define probabilities based on the angle, ridge conditions, and corner conditions to form the weights. For each position $i\in \left\{1,2,\ldots ,N_{j}\right\}$, define ridge condition weights $\alpha_{i}^{\left(R\right)}\in [0,1]$

$$\alpha_{i}^{\left(R\right)}=1_{\left\{r_{i}=1\right\}},$$

the corner condition weights $\alpha_{i}^{\left(C\right)}\in [0,1]$

$$\alpha_{i}^{\left(C\right)}=1_{\left\{c_{i}=0\right\}},$$

and the angle weights $\alpha_{i}^{\left(\theta \right)}\in [0,1]$

$$\alpha_{i}^{\left(\theta \right)}=\left(1_{\left\{\theta_{i}\in \left[\theta_{lb},\theta_{ub}\right]\right\}}\right)\alpha_{i}^{\mathrm{angfrac}},\;\mathrm{where}\;\alpha_{i}^{\mathrm{angfrac}}=\left(\frac{1}{\left|S\right|}\sum_{s\in S}\;1_{\left\{\theta_{i}^{\left(s\right)}\in \left[\theta_{lb},\theta_{ub}\right]\right\}}\right).$$

The $\alpha_{i}^{\mathrm{angfrac}}\in [0,1]$ is the fraction across scales where the angle criteria is satisfied. This factor can be removed by the *ang_frac* parameter. The final weights are constructed by multiplying the individual weights together, i.e.,

$$\alpha_{i}=\alpha_{i}^{\left(R\right)}\alpha_{i}^{\left(C\right)}\alpha_{i}^{\left(\theta \right)},\;i\in \left\{1,2,\ldots ,N_{j}\right\}$$

where the masks can be combinatorically combined by the *sum_cond* parameter (e.g. *sum_cond=”a-r”* will only multiply $\alpha_{i}^{\left(\theta \right)}$ and $\alpha_{i}^{\left(R\right)}$). *Jet saliency* is the aggregation of the weighted combination of weights $\alpha_{i}$ and the ridge strength values $\lambda_{i}$. Thus, the jet saliency for ridge $Z_{j}$ is given by

$$S\left(Z_{j}\right)=agg\left(\left\{\alpha_{1}\lambda_{1},\ldots ,\alpha_{N_{j}}\lambda_{N_{j}}\right\}\right)$$

where agg is the aggregation function, which can be the sum or the mean, depending on the *agg* parameter (see example for “sum” in Supplementary Figure S1).

#### Other metrics

MIA-Jet saves additional statistics on each ridge $Z_{j}$, namely the chromosome genomic locations, length, input mean, angle mean, width mean. Given a ridge $Z_{j}$ detected at $s^{*}\in S$ with positions $i\in \left\{1,2,\ldots ,N_{j}\right\}$,

- Start (bp): $\left(\underset{i}{min}\;x_{i}^{\prime }\right)$ (resolution), where $x_{i}^{\prime }$ are the shifted x-coordinates and *resolution* is the Hi-C bin resolution
- End (bp): $\left(\underset{i}{max}\;x_{i}^{\prime }\right)$ (resolution)
- Length (bp): $\left(N_{j}\right)$(resolution)
- Input mean: $\frac{1}{N_{j}}\sum_{i=1}^{N_{j}}I_{i}$, where $I_{i}=I\left[y_{i},x_{i}\right]$
- Angle mean: $\frac{1}{N_{j}}\sum_{i=1}^{N_{j}}\theta_{i}$, where $\theta_{i}=\Theta^{\left(s^{*}\right)}\left[y_{i},x_{i}\right]$
- Width mean: $\frac{1}{N_{j}}\sum_{i=1}^{N_{j}}w_{i}$, where $w_{i}$ is the width of ridge at point i

### Filtering

Ridges with normalized entropy $H_{\mathrm{norm}}(P)$ greater than *entropy_thresh* and normalized RMSE greater than *rmse* are removed, where *entropy_thresh* and *rmse* are user-defined parameters.

### P-Value computation

MIA-Jet assigns a p-value to each ridge based on the observed and null images $I_{\mathrm{obs}}$, $I_{\mathrm{null}}$ (Image Generation). Given a ridge $Z_{j}$, we extract the pixels of $I_{obs}$ and $I_{null}$ that lie in three boxes: the center, right and left (Figure 1 ‘Statistical test’). For the ith ridge point, $i\in \left\{1,2,\ldots ,N_{j}\right\}$, the width of each box is given by $w_{i}$ and the height is set to 1 pixel. The center box is positioned such that the ridge point $p_{i}=\left[y_{i},x_{i}\right]$ lies in the center of the box and oriented at the ridge angle $\theta_{i}$. The left and right boxes are in flanking adjacent regions to the center box (Figure 1). We extract the pixel values of each box from $I_{\mathrm{obs}}$ and $I_{\mathrm{null}}$ denoted by ${Center}_{i}^{\mathrm{obs}}$, ${Right}_{i}^{\mathrm{obs}}$, ${Left}_{i}^{\mathrm{obs}}$ and ${Center}_{i}^{\mathrm{null}}$, ${Right}_{i}^{\mathrm{null}}$, ${Left}_{i}^{\mathrm{null}}$. We then compute a local enrichment ratio for each point. For $i\in \left\{1,2,\ldots ,N_{j}\right\}$, define the observed ratio

$$T_{i}^{obs}=\frac{avg\left({\mathrm{Center}}_{i}^{obs}\right)}{avg\left(concat\left({\mathrm{Left}}_{i}^{obs},{\mathrm{Right}}_{i}^{obs}\right)\right)},$$

where concat takes a union of the two sets. Similarly, for $i\in \left\{1,2,\ldots ,N_{j}\right\}$, define the null ratio

$$T_{i}^{\mathrm{null}\;}=\frac{avg\left({\mathrm{Center}}_{i}^{\mathrm{null}\;}\right)}{avg\left(concat\left({\mathrm{Left}}_{i}^{\mathrm{null}},{\mathrm{Right}}_{i}^{\mathrm{null}}\right)\right)}.$$

We perform a one-sided hypothesis test for ridge $Z_{j}$ that $T^{obs}$ is significantly larger than $T^{\mathrm{null}}$. Let $F_{j}^{\mathrm{obs}}$ and $F_{j}^{\mathrm{null}}$ be the empirical CDFs of ${\left\{T_{i}^{\mathrm{obs}}\right\}}_{i=1}^{N_{j}}$ and ${\left\{T_{i}^{\mathrm{null}}\right\}}_{i=1}^{N_{j}}$ respectively. Then, we perform the one-sided Kolmogorov-Smirnov test to obtain the test statistic and the p-value $p_{j}^{KS}$

$$KS_{j}=\underset{t}{sup}\;\left(F_{j}^{\mathrm{null}\;}(t)-F_{j}^{\mathrm{obs}\;}(t)\right).$$

### Overlap removal

Since ridge detection is run at multiple scales of the image, there may be several overlapping ridges. We iterate through the entire set of ridges and identify overlaps, keeping only one ridge out of any set of overlaps by the lowest p-value (see Algorithm 1). Let the current set of ridges be at this stage be $Z=\left\{Z_{1},\ldots ,Z_{J}\right\}$.

**Algorithm 1:** Overlap Removal by p-Value Minimization

**Require:** Ridges $𝒵=\left\{Z_{1},\ldots ,Z_{J}\right\}$ with $p_{j}^{KS}$, j∈{1,2,…,J}; radius=7; IoUthreshold=0.05;	
**Ensure:** Indices 𝒪 of non-overlapping ridges	
1: 𝒪←∅	
2: Removed ←∅	
3: **for** each ridge index j∈{1,2,…J} **do**	
4: **if** j∈Removed **then**	
5: **continue**	
6: 𝒢←{j}	⊳ Current overlap group
7: ℰ← all ridge indices with intersection within radius of any point of $Z_{j}$	
8: **for** each t∈ℰ, (t≠j) **do**	
9: $Z_{j}^{\prime }\leftarrow buffer\left(Z_{j},\;\mathrm{radius}\right)$	
10: $Z_{t}^{\prime }\leftarrow buffer\left(Z_{t},\;\mathrm{radius}\right)$	
11: $IoU\leftarrow \frac{area\left(Z_{j}^{\prime }\cap Z_{t}^{\prime }\right)}{area\left(Z_{j}^{\prime }\cup Z_{t}^{\prime }\right)}$	
12: **if** IoU>τ **then**	
13: 𝒢←𝒢∪{t}	
14: $j^{*}\leftarrow {\arg\;min}_{t\in 𝒢}\;p_{t}^{KS}$	
15: $𝒪\leftarrow 𝒪\cup \left\{j^{*}\right\}$	
16: $Removed\leftarrow Removed\cup \left(𝒢\backslash \left\{j^{*}\right\}\right)$	
17: **return** 𝒪	

The buffer function of the *shapely.geometry* python package creates a uniform width of specified radius around a set of points. Any two ridges with intersection over union of their areas (IoU) greater than 0.05 is considered a genuine intersection. Out of the set of genuine intersections 𝒢 (which includes the current ridge j), we keep the ridge $j^{*}$ with the lowest p-value and discard the rest.

### Correction and thresholding

The Benjamini-Hochberg procedure is applied to the set of p-values $\left\{p_{j}^{KS}|j\in 𝒪\right\}$ to obtain the corrected p-values $\left\{p_{j}^{KS,\mathrm{corr}}|j\in 𝒪\right\}$. Ridges are filtered by corrected p-values with significance level provided by the *alpha* user parameter. Ridges with jet saliency less than the *saliency_thresh* percentile of jet saliency values are also filtered.

### Outputs

For each run, sub-folders are generated for each significance cutoff (*--alpha*) specified and in combination with the saliency percentile threshold (*--saliency_thresh*). Within each sub-folder, two results table are generated: a summary table (.csv) and expanded table (.csv). The summary table contains one row per chromatin jet and details quantification metrics, such as the jet saliency and p-values (Quantification). The expanded table contains the per-position information, such as genomic coordinates, angle, ridge strength, and width. Ridges are uniquely indexed by the key column “unique_id” between the two tables. A diagnostic image is generated in addition to bedpe visualization files that can be loaded into *Juicebox*.

Since MIA-Jet runs each chromosome independently, the results can be combined across chromosomes using “notebooks/combine_results.ipynb”.

### Analysis of results

#### Running Fun

The Fun pipeline (Liu et al., 2024) was run at v1.0.1. The pipeline was run with ICE normalized mcool files at the resolution specified in Supplementary Table S1 ‘Analysis Parameters’. *calculate-son-score* was run with parameters *--norm weight*, *use_mean True, --coverage_ratio 0*, --*padding_width 2*. The *--offset* parameter was set to 5 times the Hi-C resolution and *--ext_length* as floor(win-floor(win/6))/2), where win is specified in Supplementary Table S1 ‘Analysis Parameters’. *find-fontains* was run with the same parameter values as *calculate-son-score* for *--ext_length*, *--norm*, *--offset*, --*coverage_ratio*. Other parameters were specified as the following: *–half_width 2*, *--extension_pixels 10,100,5*, *--interval_length* as 5 times the Hi-C resolution, *--max_merge_distance* as 5 times the Hi-C resolution, *--p_value 0.05*, *--signal_noise_background 1.5*.

#### Running Fontanka

The Fontanka algorithm was run at v0.1. We utilized the binary fountain mask as opposed to creating a custom aggregate mask. The algorithm involves creating an expected vector from *cooltools.expected_cis*. The *view_df* parameter was set to the chromosome arms object from *bioframe.make_chromarms* with the appropriate centromeres from *bioframe.fetch_centromeres*. *fontanka slice-windows* was run with ICE normalized mcool files at the resolution specified in the config file. *-W* was set to win in Supplementary Table S1 ‘Analysis Parameters’ and *--view* with the generated chromosome arms. In cases where the bioframe centromeres were undefined for certain genomes, the entire chromosome was passed in as the chromosome arms. *fontanka apply-binary-fountain-mask* was run with *-A 0.3491, -W* was set to win, *--view* with the generated chromosome arms.

#### Processing Fontanka results

For Repli Hi-C data, we applied the maximum of the Li threshold and 0 on “FS_peaks” using *skimage.filtres.threshold_li* as in “examples/00_fontanka_zebrafish.ipynb”. For Hi-C data where TADs or loops may result in false positives, we followed the thresholding scheme in “examples/02_fontanka_xenopus.ipynb” which applied additional filtering steps. Specifically, we kept jets that were above the 90^th^ percentile of “FS_peaks” values and jets below the 75^th^ percentile of “Scharr_box” values.

#### Running MIA-Jet

The MIA-Jet algorithm was run at v1.0.20 using the resolution, norm, and exp parameters in Supplementary Table S1 ‘Analysis Parameters’. All reported results used the 90^th^ percentile jet saliency threshold and p-value threshold of 0.1 (“*results_saliency-90-p-0.1”).

#### Generating figures

Aggregate plots (Figure 2 b, f, h; Figure S2 b, c, e; Figure 3 a, b, c; Figure 4 c; Figure S4 b, e). We defined start and end genomic coordinates for each jet. The resolution of a jet region was determined by the following formula

$$\underset{\mathrm{res}\;}{min}\;\left|\frac{\;\mathrm{end}\;-\;\mathrm{start}\;}{\;\mathrm{res}\;}-100\right|$$

where res is the possible resolutions stored in the .hic file and 100 is the desired snippet size. This ensures that extracted jets (whether small or large) have sufficiently high resolution. The matrices were then resized to be 100×100 using *cv.resize* and then averaged element-wise.

Venn diagrams (Figure 2 d; Figure S2 b, e; Figure 3 b; Figure S3 b). Given two sets of jets A and B, we first stratified jets by chromosome such that we compute the intersection of jets for one chromosome at a time and later summed genome wide. Since jets are two-dimensional objects which can intersect with multiple other jets, we first compute the intersection over union IoU of A and B in a “left join” fashion: every jet in A records if it has any intersection(s), potentially multiple, with the jets in B, recording the jet index in B as well as the IoU of each intersection. Similarly, we then compute the IoU of A and B in a “right join” fashion. This results in a weighted, directed graph between the jets in set A and the jets in set B, where the nodes are jets and weights are given by the IoU values. Here, we seek an optimal pairing (i.e. one-to-one matches) between the jets in A and B. We use the *max_weight_matching* function from the *NetworkX* python package to assign these pairs. Finally, we simply count the number of paired and non-paired, where the number of paired nodes is the common intersection and the number of non-paired in each A and B give the A only and B only respectively.

Boxplots (Figure 2 c, d; Figure 3 d; S3 c). ChIP-seq data, insulation scores, compartment scores, and repli-seq data was loaded using the *stats* function from *pyBedGraph* python package with *stat=”max”* at 10 kb resolution. The jet intervals consisted of the start and end regions being ±10kb, where the jet midpoint mp is defined as the projection of the closest position of the jet to the main diagonal. The “Observed” locations in Figure 3 d and S3 c were the MIA-Jet results. The “Random” set was generated by the *random* function from *pybedtools* with the length (l) parameter as the median of the MIA-Jet jets and the number (n) as the number of MIA-Jet jets. The random generation was stratified by chromosome and combined.

#### Defining GM12878 cohesin loading sites and anchor sites

As means of characterizing the jets identified by MIA-Jet with respect to cohesin loading and anchoring sites, 10239 cohesin-specific peaks from GM12878 cell line are further filtered by the criteria that NIPBL binding (of GSM2443453 downloaded track) is greater than 5 counts, yielding 5,467 regions. A total 3,014 convergent loops include 1 or more of these cohesin loading regions; since convergent loops may overlap (e.g., a bigger loop enclosing a smaller loop), some loading regions are recorded for two different convergent loops. These 3014 loops enclosed 8777 loading regions. We only kept loading sites that are within 25 percentile to 75 percentile from both left and right anchors, to exclude those near the anchors. The result is 2226 loops containing 4938 loading sites for GM12878 cells, but some loops have multiple loading sites and a loading site may be encapsulated inside multiple loops. As a final filter, we retained only 2352 unique loading sites and 3079 unique anchoring sites, which are used for overlapping MIA-Jet jets midpoint location extended both sides by 10 kb or random regions with bedtools intersect -wa -u.

#### CTCF loop encapsulation in GM12878 cells

The GM12878 CTCF ChIA-PET loops identified by ChIA-PIPE are further processed to extract highly confident convergent loops as follows. Loops with binding intensity greater than 250 (read counts) on both loop anchors with PET counts greater than 9 are considered strong loops. The loop anchors are annotated with CTCF motifs identified STORM as presented in Tang et al., Cell, 2015, and only the loops with convergent CTCF binding motifs are retained to annotate whether the jet falls within the two anchors (‘CTCF loop encapsulated’) or not (‘None’).

#### Annotating GM12878 jets with chromatin states

MIA-Jet jets midpoint location extended both sides by 10 kb or random regions are initially overlapped against chromHMM states to obtain all states and basepairs of overlaps in each region via bedtools intersect -wao. The chromatin states 1,2,3 are grouped into ‘promoters’, 4,5,6,7 into ‘enhancers’, 9,10,11 into ‘transcribed states’, while 8 is ‘insulator’, 12 is ‘repressed’, 13 is ‘heterochromatin’, and the rest is classified as ‘other’. Given the number of basepairs occupied by each of these 6 simplified states, if the promoter is larger than 0 bp, then the state is ’prom’. If the region does not contain any promoters and the enhancer is larger than 0 bp, then the state is ‘enh’; otherwise, the state is assigned to the one with maximum occupancy in terms of basepairs.

## Supplementary Material

Supplement 1 **Supplemental Table S1.** Summary of 3C data and auxiliary data used for analysis of MIA-Jet results. Recommended parameters for MIA-Jet and parameter settings used in this study. Related to Figure 1.

Supplement 2

## Data Availability

The MIA-Jet software is available under the MIT License: https://github.com/minjikimlab/miajet.
