# Supplementary material for: MIA-Jet: Multi-scale Identification Algorithm of Chromatin Jets
Source: bioRxiv. 2025 Sep 1:2025.08.27.672730. Preprint. [Version 1] doi: 10.1101/2025.08.27.672730 (PMC12424625; doi:10.1101/2025.08.27.672730)

bioRxiv preprint doi: https://doi.org/10.1101/2025.08.27.672730; this version posted September 1, 2025. The copyright holder for this preprint (which was not certified by peer review) is the author/funder, who has granted bioRxiv a license to display the preprint in perpetuity. It is made available under aCC-BY 4.0 International license.

SUPPLEMENTARY FIGURE TITLES AND LEGENDS

Figure S1: Detailed modules of MIA-Jet. Related to **Figure 1**.

The MIA-Jet pipeline takes a .hic image as input to generate an image. Insets show ridge characterization and quantification. Each blurred image is fed into (1) a ridge detection module which identifies ridge positions $i \in \{1, 2, \ldots, \}$ and widths and (2) a feature generation step. The feature matrices are combined across scales to form 3 dimensional tensors, plotted such that the z-axis represents each scale. These tensors are used for both trimming (see **Methods**) and quantification, where three distinct metrics are computed for each ridge. 'Normalized entropy' extracts ridge strength λ values along the coordinates and computes the mean ridge strength $\bar{\lambda}$. This is binned into a histogram to compute its normalized entropy, where β ensures that the entropy is in $[0,1]$. False positives are expected to have large entropy. 'Normalized RMSE' extracts ridge strength λ values along the ridge position to compute the expected scale $\bar{s}$, which for this example, captures the gradual diffuseness of the chromatin jet by an increasing function. The normalized root mean squared error to a cubic fit serves as a reasonable metric to quantify realistic scale profiles. 'Jet saliency' extracts the slice at scale s^*, which is the scale at which the current ridge was detected. Along the ridge positions, probabilities (red) are computed which indicate whether specific conditions are satisfied, namely that the current point i is in the right angle, is a ridge, and not a corner. These probabilities are combined in a weighted sum of ridge strength values to rank ridges. Examples show MIA-Jet results in mouse DP thymocyte Hi-C data in the top 10, 90th percentile, 75th percentile, and bottom 10 (from left to right).

Figure S2: Examples and analyses of jets in DP thymocytes Hi-C, Splenic B cells Hi-C, and K562 Repli-HiC data. Related to **Figure 2**.

(a) Top 5 jets identified by MIA-Jet, Fun, and Fontanka in DP thymocytes Hi-C data with reference genome mm10. Hi-C contacts shown observed/expected counts ('oe'). **(b)** Left: A Venn diagram of jets between DP thymocytes and Splenic B cell Hi-C data, where n denotes the number of jets in each category. Right: aggregation of 2D contacts at three categories of jet regions. **(c)** Using 142 jet regions identified in DP thymocytes, Hi-C aggregation of both DP thymocytes and Splenic B cells. **(d)** Similar to panel **(a)** but for K562 Repli-HiC data. **(e)** Comparison of jets identified by MIA-Jet, Fun, and Fontanka, in K562 Repli-HiC data with Venn diagram on the left and 2D contact aggregation on the right. **(f)** Histograms of jet length distributions of jets identified by MIA-Jet (left) and Fun (right) in K562 Repli-HiC. The number of jets is reported as n, alongside median and mean. **(g)** Bar charts of run time (in minutes) of MIA-Jet, Fun, and Fontanka on DP thymocytes

bioRxiv preprint doi: https://doi.org/10.1101/2025.08.27.672730; this version posted September 1, 2025. The copyright holder for this preprint (which was not certified by peer review) is the author/funder, who has granted bioRxiv a license to display the preprint in perpetuity. It is made available under aCC-BY 4.0 International license.

Hi-C (left) and K562 Repli-HiC (right) data. **(h)** Bar charts of memory usage (in GigaBytes) of MIA-Jet, Fun, and Fontanka on DP thymocytes Hi-C (left) and K562 Repli-HiC (right) data.

Figure S3: Characterization of jets identified by MIA-Jet in K562 and GM12878 cell lines. Related to **Figure 3**.

(a) A summary table of the median and mean of jet lengths identified by MIA-Jet in K562 cell line. **(b)** Venn diagrams of jets identified in pairs of K562 Repli-HiC, in situ Hi-C, and intact Hi-C. n: number of jets **(c)** A table of the median and mean of jet lengths identified by MIA-Jet in GM12878 cell line. **(d)** The number of jets overlapping between two datasets in the lower left triangle, and jaccard index in the upper right triangle. **(e)** Boxplots of max insulation scores in ±10 kb regions of observed jets and random locations across GM12878 technologies. **(f)** Boxplot of max compartment scores in ±10 kb regions at same locations as panel (e) **(g)** Boxplots of max NIPBL ChIP-seq in ±10 kb regions with log(signal + 1) transformation **(h)** Boxplots of log2 fold change of early and late repli-seq signal. Signal was extracted by taking the max of ±10 kb regions **(i)** Stacked bar chart of jets associated with cohesin loading (yellow), CTCF anchoring (blue), or other regions for MIA-Jet jet locations and Random locations. **(j)** The number of jets (top) or random regions (bottom) encapsulated by convergent CTCF loops (‘Loop encap.’; blue) or not encapsulated by it (‘None’; grey). **(k)** Composition of chromHMM chromatin states at jet origins (see **Methods**).

Figure S4: Analysis of jets identified in protein-depleted cells. Related to **Figure 4**.

(a) Similar to **Figure 4a** in a different region. **(b)** Aggregation of 2D contacts at 111 jet regions identified in C. elegans control Hi-C data, for control, SMC3-depleted, and WAPL-depleted cells. **(c)** A 100 kb region at 500 bp resolution in C. elegans control, SMC3-depleted, and WAPL-depleted cells Hi-C data with MIA-Jet annotations in green (if detected). **(d)** The 2D contact aggregation of zebrafish embryo Hi-C jets, with n denoting the number of jets identified by MIA-Jet. **(e)** Two exemplary locations of straight (left) and curved (right) jets with MIA-Jet widths in green with contact map at 25 kb resolution.

bioRxiv preprint doi: https://doi.org/10.1101/2025.08.27.672730; this version posted September 1, 2025. The copyright holder for this preprint (which was not certified by peer review) is the author/funder, who has granted bioRxiv a license to display the preprint in perpetuity. It is made available under aCC-BY 4.0 International license.

Figure S1

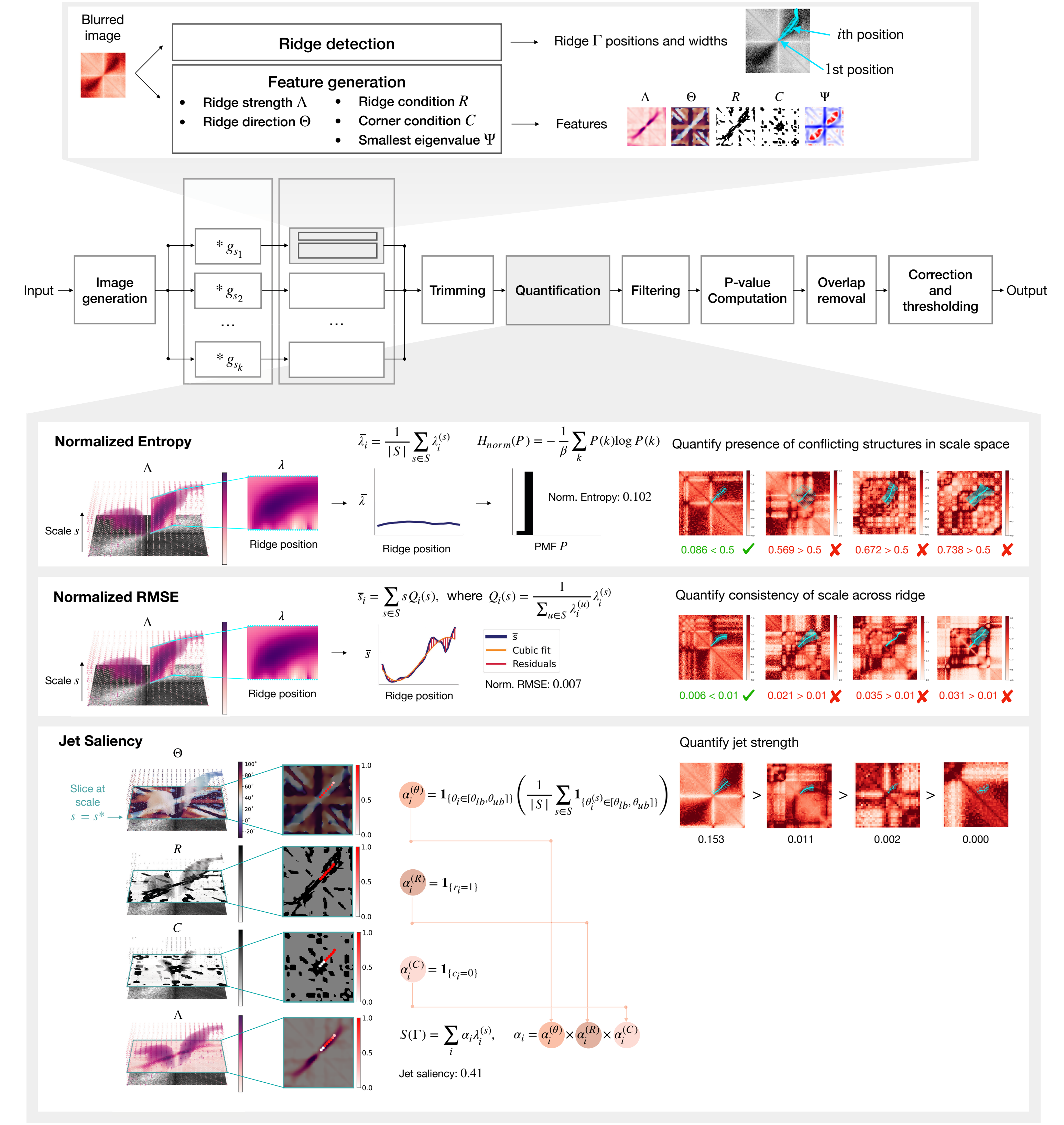

bioRxiv preprint doi: https://doi.org/10.1101/2025.08.27.672730; this version posted September 1, 2025. The copyright holder for this preprint (which was not certified by peer review) is the author/funder, who has granted bioRxiv a license to display the preprint in perpetuity. It is made available under aCC-BY 4.0 International license.

Figure S2

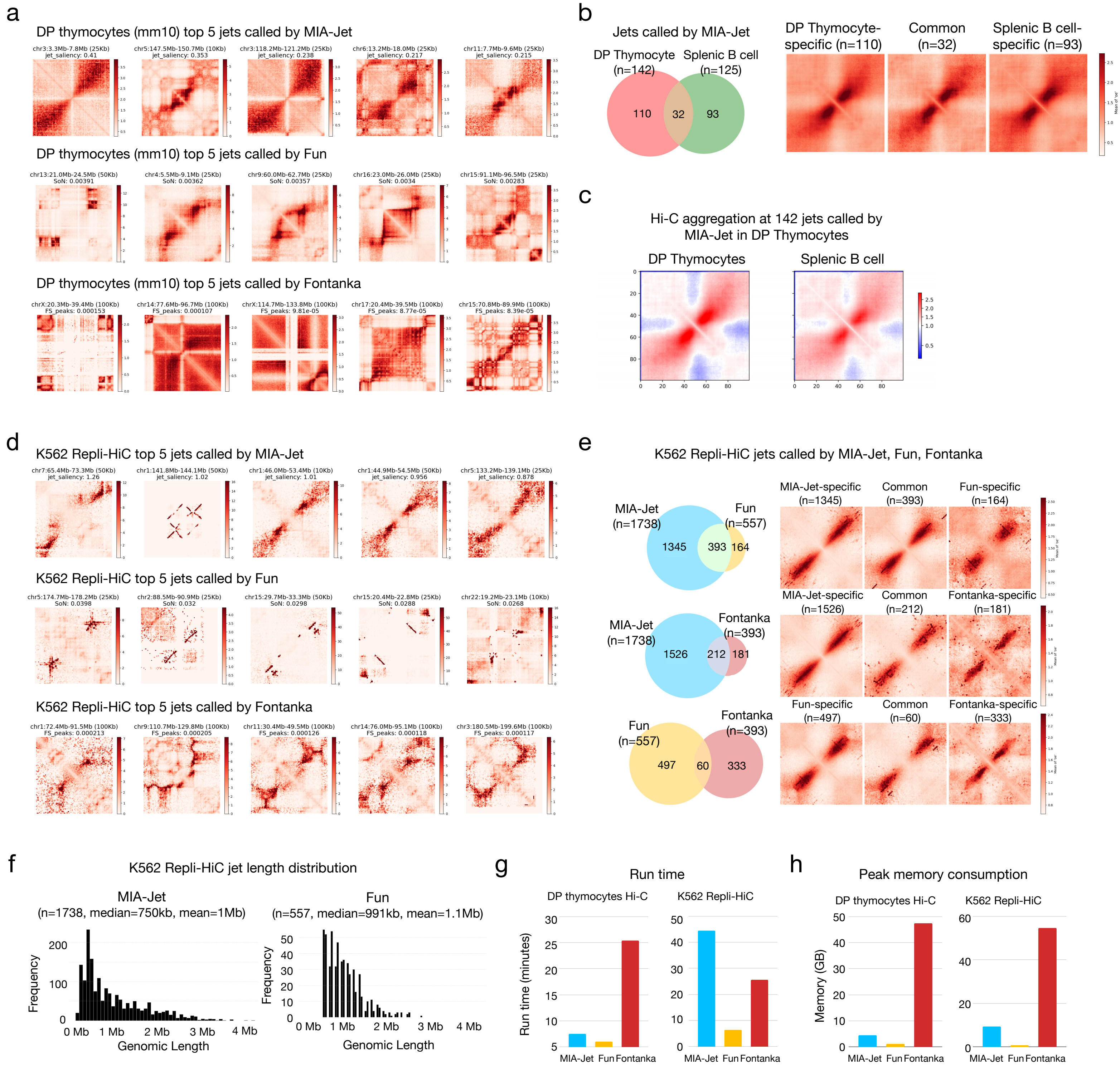

bioRxiv preprint doi: https://doi.org/10.1101/2025.08.27.672730; this version posted September 1, 2025. The copyright holder for this preprint (which was not certified by peer review) is the author/funder, who has granted bioRxiv a license to display the preprint in perpetuity. It is made available under aCC-BY 4.0 International license.

Figure S3

a

K562 jet length distribution

K562	# of jets	Median	Mean
**Repli-HiC**	1738	750 kb	1 Mb
**In situ Hi-C**	219	500 kb	547 kb
**Intact Hi-C**	269	500 kb	612 kb

b

Jets called by MIA-Jet in K562 Repli-HiC, in situ Hi-C, intact Hi-C

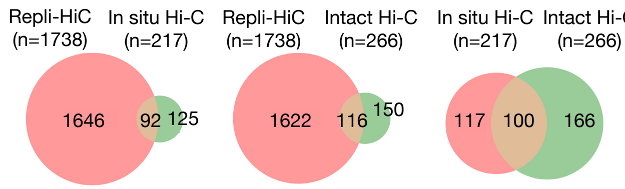

c

GM12878 jet length distribution

GM12878	# of jets	Median	Mean
**In situ Hi-C**	277	750 kb	562 kb
**Intact Hi-C**	281	500 kb	596 kb
**RAD21 ChIA-PET**	201	350 kb	412 kb
**RNAPII ChIA-PET**	151	300 kb	362 kb
**RNAPII ChIA-Drop**	202	300 kb	360 kb

d

Jet overlaps between two datasets

GM12878	In situ Hi-C	Intact Hi-C	RAD21 ChIA-PET	RNAPII ChIA-PET	RNAPII ChIA-Drop
**In situ Hi-C**	277	0.325	0.216	0.163	0.151
**Intact Hi-C**	137	281	0.199	0.137	0.145
**RAD21 ChIA-PET**	85	80	201	0.262	0.229
**RNAPII ChIA-PET**	60	52	73	151	0.221
**RNAPII ChIA-Drop**	63	61	75	64	202

e

Insulation Scores

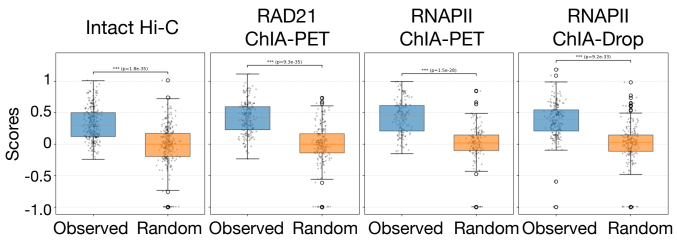

f

Compartment score

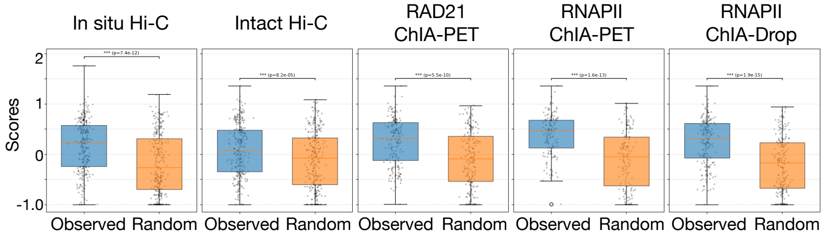

g

NIPBL ChIP-seq

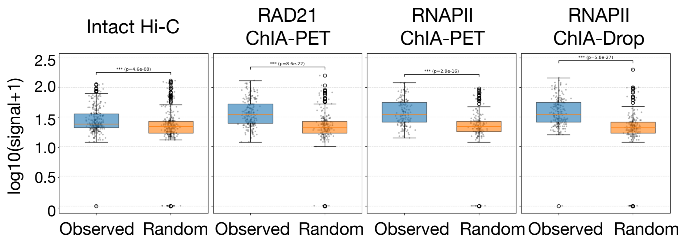

h

Early/late replication

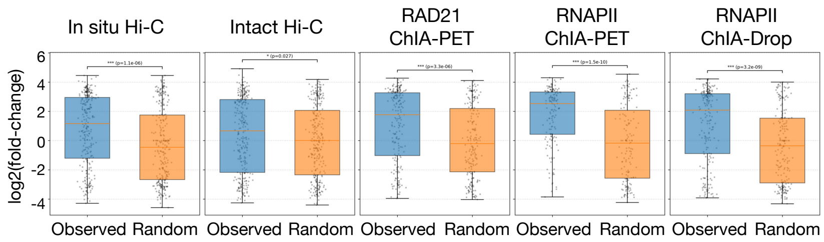

i

GM12878 in situ Hi-C loading vs. anchor

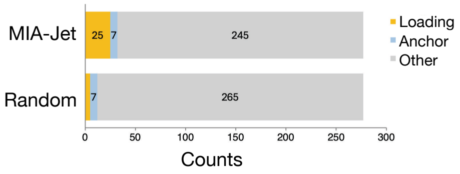

j

GM12878 in situ Hi-C CTCF encapsulation

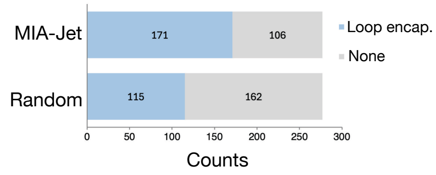

k

GM12878 in situ Hi-C ChromHMM states

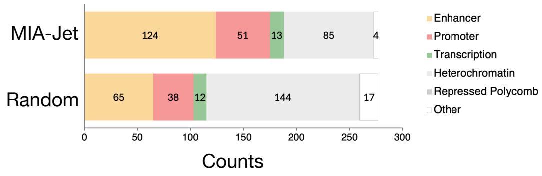

bioRxiv preprint doi: https://doi.org/10.1101/2025.08.27.672730; this version posted September 1, 2025. The copyright holder for this preprint (which was not certified by peer review) is the author/funder, who has granted bioRxiv a license to display the preprint in perpetuity. It is made available under aCC-BY 4.0 International license.

Figure S4

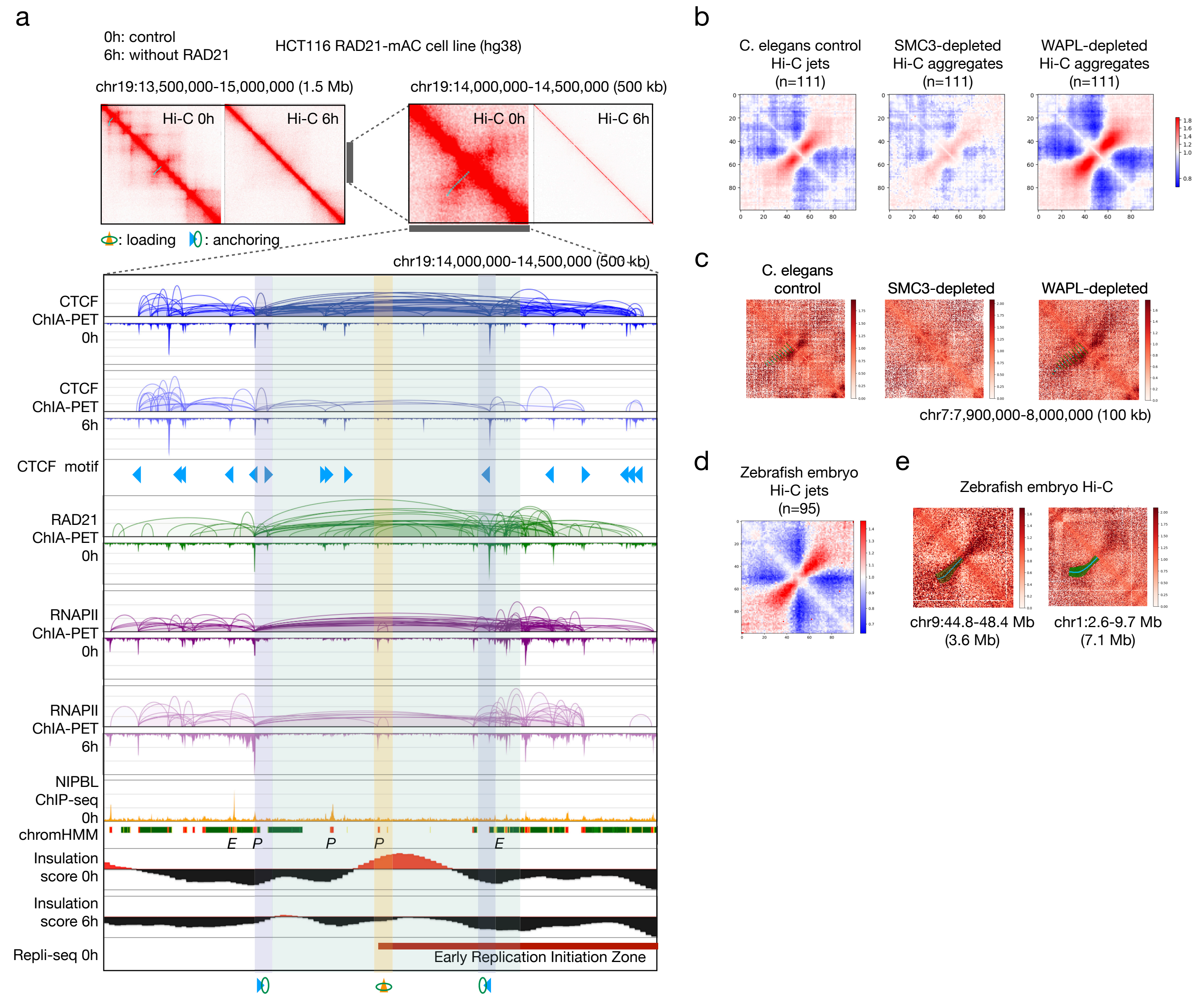

Supplement: Supplement 2 [file NIHPP2025.08.27.672730v1-supplement-2.pdf]
